# On the Kinematics of the Forward-Facing Venetian-Style Rowing Technique

**DOI:** 10.3390/bioengineering10030310

**Published:** 2023-02-28

**Authors:** Joseph N. Grima, Dario Cerasola, Anabel Sciriha, Darren Sillato, Cynthia Formosa, Alfred Gatt, Michael Gauci, John Xerri de Caro, Robert Needham, Nachiappan Chockalingam, Tonio P. Agius

**Affiliations:** 1 Metamaterials Unit, Faculty of Science, University of Malta, MSD 2080 Msida, Malta; 2 Siġġiewi Rowing Club, 181, Melita Street, VLT 1129 Valletta, Malta; 3 Italian Rowing Federation, Viale Tiziano, 74, 00196 Rome, Italy; 4 Department of Psychology, Educational Science and Human Movement, University of Palermo, 90100 Palermo, Italy; 5 Department of Physiotherapy, Faculty of Health Sciences, University of Malta, MSD 2080 Msida, Malta; 6 Department of Podiatry, Faculty of Health Sciences, University of Malta, MSD 2080 Msida, Malta; 7 Centre for Biomechanics and Rehabilitation Technologies, School of Health, Science and Wellbeing, Staffordshire University, Stoke-on-Trent ST4 2DF, UK

**Keywords:** rowing, venetian rowing, kinematics, sports biomechanics, traditional rowing, motion analysis

## Abstract

This work presents a qualitative and quantitative pilot study which explores the kinematics of Venetian style forward-facing standing rowing as practised by able-bodied competitive athletes. The technique, made famous by the gondoliers, was replicated in a biomechanics laboratory by a cohort of four experienced rowers who compete in this style at National Level events in Malta. Athletes were marked with reflective markers following the modified Helen Hayes model and asked to row in a manner which mimics their on-water practise and recorded using a Vicon optoelectronic motion capture system. Data collected were compared to its equivalent using a standard sliding-seat ergometer as well as data collated from observations of athletes rowing on water, thus permitting the documentation of the manner of how this technique is performed. It was shown that this rowing style is characterised by rather asymmetric and complex kinematics, particularly upper-body movements which provides the athlete with a total-body workout involving all major muscle groups working either isometrically, to provide stability, or actively.

## 1. Introduction

Rowing, one of the oldest sports that form a part of the Olympic program, is generally practised with the athlete seated facing the rear of the direction of travel. Other rowing styles exist, such as the mode popularised by the gondoliers in Venice in which the athlete is not seated, but standing and forward facing [1,2]. The latter style is widely practised as a competitive sport in various regions of Italy, where it is referred to as “*Voga Veneta*”, “*Voga alla Veneziana*” (Venetian rowing) or “*Voga in Piedi*” (standing rowing), and in other parts of the world. Compared to classic “traditional” rowing where the rower sits on fixed-seat boats, or the more biomechanically efficient “Olympic-type rowing” where the seat is allowed to slide, standing rowing offers the advantage that the athletes can easily steer the boat without needing to look backwards, or to make use of a coxswain. This makes it considerably safer to practise, particularly in harbours or similarly busy waters, and permits the athletes to focus on rowing as an exercise rather than concentrating on how to avoid collisions. 

**Figure 1:**
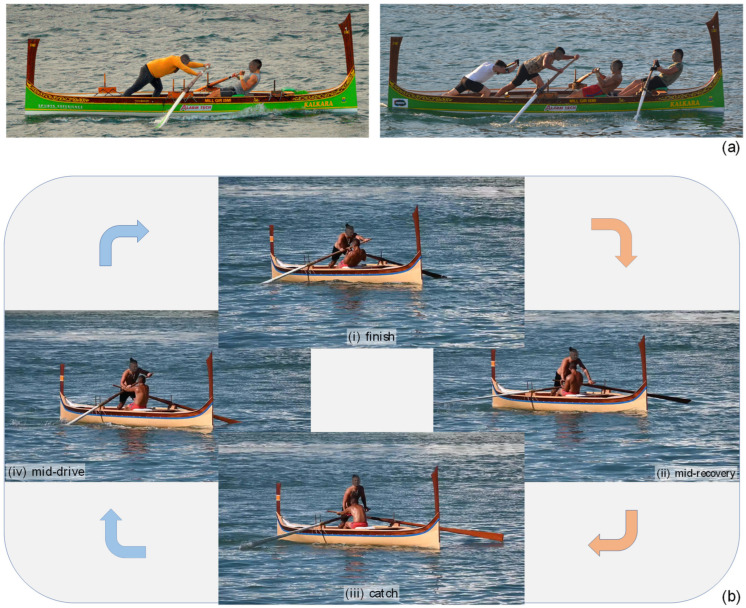
The basics of Venetian style standing rowing, demonstrated here on the Maltese “*Dgħajsa tal-Pass*” racing traditional rowing boat where the standing rower is shown alongside a seated rowing. (**a**) A shows the “finish” position for the boat set up with a crew of two (**left**) or four (**right**) rowers whilst (**b**) highlights the key components of the cycle, where a standing rower (locally known as the “*parasija*”) is rowing in sync with a standard traditional seated rower (locally known as “*irmiġġier*”). Illustrated here are, approximately, (**i**) the finish, (**ii**) mid-recovery, (**iii**) the catch and (**iv**) mid-drive. Note that in boats with two rowers, the seated rower keeps the stoke rate whilst the standing rower steers.

A European nation where standing Venetian-style rowing is widely practised as a sport is the Republic of Malta, a country neighbouring Italy. Here, a historical National Regatta rowed on traditional wooden boats over a distance of slightly over 1 km, which traces its origin to the 17th century, sees most of the competing boats rowed by a combination of sitting and standing rowers. Such crews are composed of two or four athletes where, as shown in Figure 1a, the standing rowers stand on the rear part of the boat, facing the sitting rowers who sit on fixed seats in the front. The basic movements of this technique, illustrated in Figure 1b, require standing rowers to essentially “push” the oar in the active “drive phase”, from the “catch” to the “finish”, in synchrony with the “pulling” actions of seated rowers (and vice versa in the “recovery phase”). 

Although traditional seated rowing, Olympic-style rowing as well as indoor rowing (i.e., rowing on an ergometer (erg), a simulated form of rowing used for training and competitions, which doubles as a very useful tool for studying sliding-seat rowing within a controlled laboratory setting) have been studied extensively from various perspectives [3,4], ranging from biomechanics [5,6,7,8,9,10,11,12,13,14], physiology [15,16], strategy [17,18,19,20,21], and injuries [22,23,24,25,26,27,28], the scientific literature regarding the sporting practise of Venetian-style standing rowing remains scarce [1,2], particularly from the aspect of biomechanics, despite the long tradition and the relative popularity of this sport. This work aims to address this lacuna by documenting, for the first time, the rather complex kinematics of the forward-facing Venetian Rowing Technique (VRT), which have been recorded using calibrated motion-capture equipment in a laboratory setting.

## 2. Materials and Methods

### 2.1. Participants

Four oarsmen (referred to as Rower 1, 2, 3 and 4), all male, having an average age of 24.5 ± 2.6 years, average height 180.0 ± 8.0 cm, average weight 94.5 ± 11.0 kg and average body mass index (BMI) of 30.0 ± 3.7, who row in the Maltese National Regatta in the standing *parasija* positions (i.e., in Venetian-style, with the oar on their right), volunteered to participate in this study. Rowers 1 and 3 were of similar height and BMI, taller than the other two rowers who also had similar height but very different BMI (Rower 2 had BMI of 26, Rower 4 had BMI of 35, compared to Rowers 1 and 3 who had a BMI of 29 and 30 respectively). The chosen inclusion criteria for this study were that the participants:

i. had to have been rowing for at least three years in this form;
ii. were above the age of 18;
iii. did not suffer from any acute musculoskeletal injuries or other comorbidities e.g., cardiorespiratory issues.

All participants fulfilled these criteria and exceeded them in terms of rowing experience. The four participating rowers had been rowing since their childhood, were regular participants in the Maltese National Regattas at the time of the study and had won several medals at national level in their rowing careers. 

Participants were pre-informed about the procedure verbally and through a detailed information letter, and voluntarily agreed to participate in the study. All participants were informed that the procedure would be non-invasive and would pose no different risk to those they were accustomed to in their sport training. Their intention to participate in this study was signified through signing of an informed consent form that was previously approved by the Faculty Research and Ethics Committee (Approval number: FREC FHS_1718_017). Informed consent was also obtained to publish the information/images in an online open access publication.

### 2.2. Protocol Used

**Figure 2:**
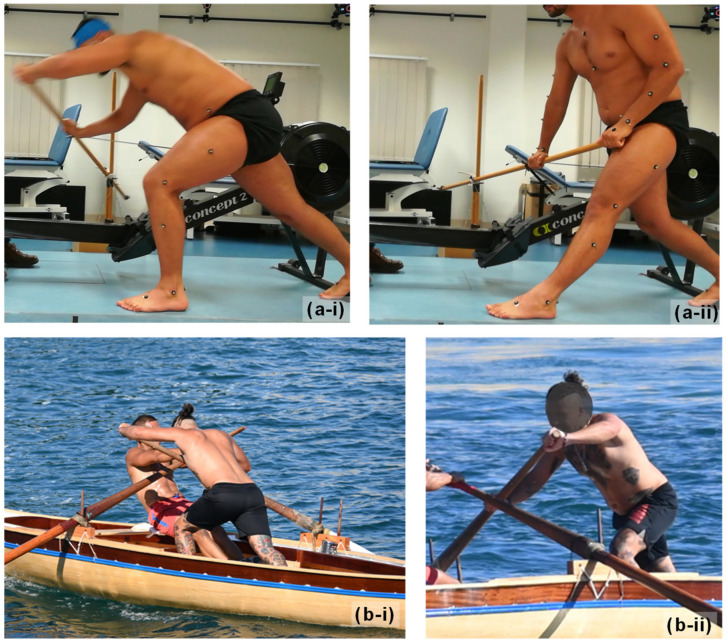
(**a**) The set up used in the laboratory to mimic the on-water setup, shown in (**b**). Note that the rowers in (**a-i**) and (**b**) are nearing to the “finish” whilst the rower in (**a-ii**) is nearing the “catch”. Note also the position of the reflective markers, shown more clearly in (**a-ii**).

Standing Venetian-style rowing, as practised by athletes in Malta, was simulated using a standard Concept2 Model D rowing ergometer modified as in Figure 2a to be able to replicate Venetian-style standing with the oar on the right-hand side of the rower. This setup was designed and constructed in a manner which closely mimicked the boat’s interior, both in terms of size and rowing experience. Key to this design were: (i) the ergometer itself was able to reproduce a sensation of resistance as felt by the athletes when rowing on-water; (ii) a vertical pole with a horizontal stopper, which was set at the appropriate height and separation from the rower to replicate the oar-lock position; (iii) a wooden stick to replicate the oar, replacing the ergometer handle as shown in Figure 2a, which was held by the rower on one side, and affixed near its other end to the aforementioned oar-lock replica; (iv) a rope which was knotted in a manner which replicated the normal oar-lock knot used on boats. Note that in this case, since Venetian rowing is essentially a “pushing” action, rather than “pulling”, as is the case in conventional seated rowing, the ergometer had to face in the opposite direction the rower faced. Moreover, the ergometer chain had to be attached to the oar and positioned between the rower and the oar-lock, such that it did not interfere with the rower’s movements. 

Data collection was conducted in the Biomechanics Research Laboratory at the Faculty of Health Sciences, University of Malta using a Vicon optoelectronic motion-capture system (Oxford metrics, Oxford, UK) comprised of 16-cameras hung on solid brackets below the ceiling, operating at a sample rate of 100 Hz. This “Optical-Passive” setup could record the position of retroreflective markers placed on individuals that are tracked using 16 infrared cameras, which were calibrated using a standard calibration protocol. Athletes’ movements were recorded inside this calibrated volume and the Vicon Nexus^®^ software version 2.8.1 (Oxford metrics, Oxford, UK) was used to record the coordinates and to generate a set of angular measurements related to the joints performing the movements.

On the day of the experiments, participants were first asked to familiarise themselves with the laboratory setup so that they could give feedback on its ability to adequately replicate the real boat setup. They were then marked with 39 spherical reflective markers following the modified Helen Hayes model (PlugIn Gait, Vicon, Oxford, UK), see Figure 2(a-ii), the position of which was detected using the 16-camera setup, and asked to test the setup again and warm up in their preferred manner. 

Participants were then asked to row on this setup at a self-selected power and stroke rate (fairly common in gait analysis [29]), and the three-dimensional movements of the trunk, pelvis, upper and lower limbs were sampled over four 10 s capture with these being recorded at randomly selected periods during the experiment. Data extracted from this protocol included:

(a) Angles which measure the orientation of the thorax (Thorax1; Thorax2 and Thorax3) and pelvis (Pelvis1; Pelvis2 and Pelvis3) relative to the global axis, as well as spine angles (Spine1; Spine2 and Spine3) which relate with the measurements of the thorax and pelvis angles, relative to each other;
(b) Angles which relate to the lower limbs, namely the hip (Hip1; Hip2 and Hip3), knee (Knee1) and ankle (Ankle1) angles;
(c) Angles which relate to upper limbs, namely the shoulder angles (Shoulder1; Shoulder 2 and Shoulder 3) and Ankle (Ankle 1) angles.

where the numerals ‘1’, ‘2’ and ‘3’ refer to the measurement being in the sagittal, coronal, and transverse plane, or its equivalent. 

### 2.3. Analysis

Three full rowing cycles (strokes), starting from the “finish” (i.e., from the end of the drive phase / start of the recovery), for each of the four rowers (i.e., twelve cycles in total) were selected for further analysis and synchronised in a manner that the “catch” and finish” were made to correspond and then plotted as a percentage of the cycle. 

Moreover, for each of these twelve standing rowing cycles, and for each angular measurement (*θ* made, the following key data were noted and further analysed statistically to obtain the mean and standard deviation for each angular parameter:

*θ*_max_ the maximum value of the angle *θ* attained within the cycle
*θ*_min_ the minimum value of the angle *θ* attained within the cycle
*θ*_ROM_ the range of motion associated with the angular measurement *θ* where *θ*_ROM_ = *θ*_max_ − *θ*_min_

This data were also compared to equivalent data for rowing on a standard sliding-seat ergometer obtained from a cohort of nine rowers, total number of rowing cycles analysed *n* = 3 × 8 = 24 (average age: 23.9 ± 2.6 years, average height 177.6 ± 6.8 cm, average weight 89.1 ± 9.9 kg, all fixed-seat competitive rowers who also trained and/or competed using the standard sliding-seat rowing ergometer.). Analyses through Inferential statistics using the Statistical Package for Social Science (SPSS^®^) software, version 25.0 (IBM Corp., Released 2017, Armonk, NY: IBM Corp.), was carried out on these angular parameters using the Mann–Whitney U-test. This test was chosen as a test for normality of the parameters *θ*_max_, *θ*_min_ and *θ*_ROM_ using the Shapiro–Wilk test suggested that the data were not normally distributed.

### 2.4. Comparison with On-Water Rowing

Apart from the data collected in the laboratory, additional data were also collected from on-water rowing during training in the period just before a regatta. Rowers were observed in a non-intrusive manner, to negate the well-known Hawthorne and Rosenthal effects, from a public vantage point, located in the second part of the normal racing route, that is typically reserved for spectators and photographers. The rowing action was recorded using a digital camera (Nikon D3200 DSLR camera with a Sigma lens (150–600 mm), or a Nikon DSLR Camera D750, with a Sigma 70–200 f/2.8) at a high enough zoom-level where the movements of individuals were clearly and sharply identifiable, ensuring availability of around two to four cycles to have at least one complete cycle for analysis. Recording was performed 2–3 m above sea-level with the camera pointed perpendicular to the racing route to minimise perspective or parallax errors. Images were extracted from the respective clip at an appropriate frame rate and manually reviewed to choose the optimal captures. The coordinates of key points which described the actions of the rowers were identified from these captures through an in-house written script which outputs the pixel numbers of selected positions. Measurements from these coordinates were then used to verify the shape of the trends obtained quantitatively in the laboratory, e.g., findings related to trunk orientation. 

### 2.5. Reporting

The kinematic data collected in the laboratory were reported through a combination of sequences of images, as well as plots which provides a graphical report of the manner of how the angular parameters vary, from finish to finish as a percentage of the cycle, analogous to earlier work related to standard (sliding-seat ergometer) rowing [12] and gait analysis (reported as a percentage of gait cycle) [30]. Note that in these plots these angular parameters were reported as the averaged results ±1.96 standard deviations to provide an indication of the spread of data points, even if, given the small sample size of only four participants in the standing rower study, the assumption of normality of the data may not be true. In fact, a test for normality of the parameters *θ*_max_, *θ*_min_ and *θ*_ROM_ using the Shapiro–Wilk test suggested that the data were not normally distributed. Various angle–angle graphs are also plotted to help provide a better appreciation of the kinematics of standing rowing. 

## 3. Results

**Figure 3:**
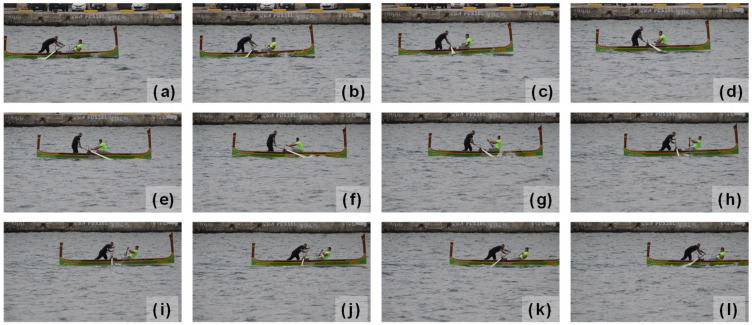
A detailed sequence of images showing the kinematics of standing rowing recorded on water on a Maltese *Dgħajsa tal-Pass*. Note that the catch, drive, finish and recovery of the sitting and standing rowers is synchronised, as is normal in rowing. (**a**–**l**) illustrate different parts of the stroke with (**a**,**l**) representing the finish; with the catch commencing at (**e**).

**Figure 4:**
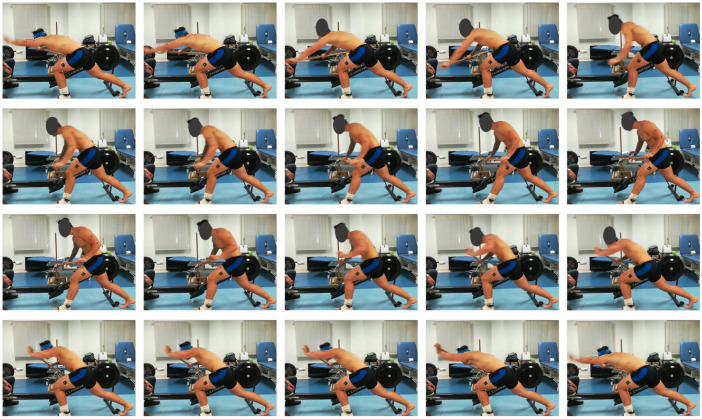
A sequence of images showing a typical standing rowing cycle as performed in the lab, with the first two rows of images showing the “recovery” phase (starting from the “finish” position) and the last two rows showing the “drive” phase (starting from the “catch”, ending with the “finish”).

A sequence of photographic images showing a typical full stroke (rowing cycle) of a standing (*parasija*) and sitting athlete (*irmiġġier*) rowing the traditional *Tal-Pass* Maltese boat are shown in Figure 3, whilst a second sequence of photographic images showing a typical subject undergoing this experiment in the laboratory are shown in Figure 4.

Examining first the on-water sequence of images, it should first and foremost be noted that standing Venetian style rowing cycle can also be described in terms of an active “drive” phase (Figure 3f–l) and a “recovery” phase (Figure 3a–e), which is essentially a reverse of the drive. Given that, as on any boat, the “catch” and “finish” of the two rowers need to be perfectly synchronised timewise to ensure the boat moves efficiently, one can look at the movement of the sitting rower to help interpret the kinematics of the standing rower, a feature which makes Malta an ideal location for this present study. As evident in these images, the standing rower “pushes” the oar in the active drive phase, accompanying the “pulling” action of the sitting rower, to take the oar from the “catch” (Figure 3f) to the “finish” (Figure 3l/Figure 3a), whilst in the recovery phase, the standing rower “pulls” the oar, accompanying the “pushing” action of the sitting rower. Of note is that a coxswain was not required as the standing rower faces the forward direction takes the role to steer the boat. 

A comparison of images in Figure 3 with those in Figure 4, both involving a different subject, suggests that the on-water kinematics were very well replicated in the laboratory setup. Figure 4 depicts the “recovery” phase in the first two rows of images and the “drive” phase in the last two rows. 

Moreover, as illustrated in these two sets of images (Figure 3 and Figure 4), a number of qualitative inferences, which are discussed in more detail in the next section, could be made, including:

i. The kinematics of standing rowing are rather asymmetric, and involve complex movements, particularly upper-body movements;
ii. Standing rowing provides the athlete with a total-body workout involving all major muscle groups working either isometrically, to provide stability, or actively;
iii. The energy input of the sitting and standing rower to propel the boat seem, to a first approximation, to be not too dissimilar from each other, as discussed further below.

Moreover, quantitative results of angular measurements taken in the laboratory are reported as graphs showing the variation of the angular measurements with a percentage of the cycle, finish to finish and in tables which report the mean and standard deviations of *θ*_ROM_, *θ*_min_ and *θ*_max_. These measurements, as discussed in more detail below, confirm the complexity and asymmetricity of standing rowing, which is very distinct from that of standard seated rowing. The tables presented contain information related to each of the four rowers studied, the average of the four rowers, and a comparison with the respective average values measured for standard sliding-seat ergometer rowing (together with the respective *p*-values comparing the average measurements for standing rowing and standard sliding-seat ergometer rowing). 

### 3.1. Measurements Related to the Kinematics of the Thorax, Pelvis and Spine

**Figure 5:**
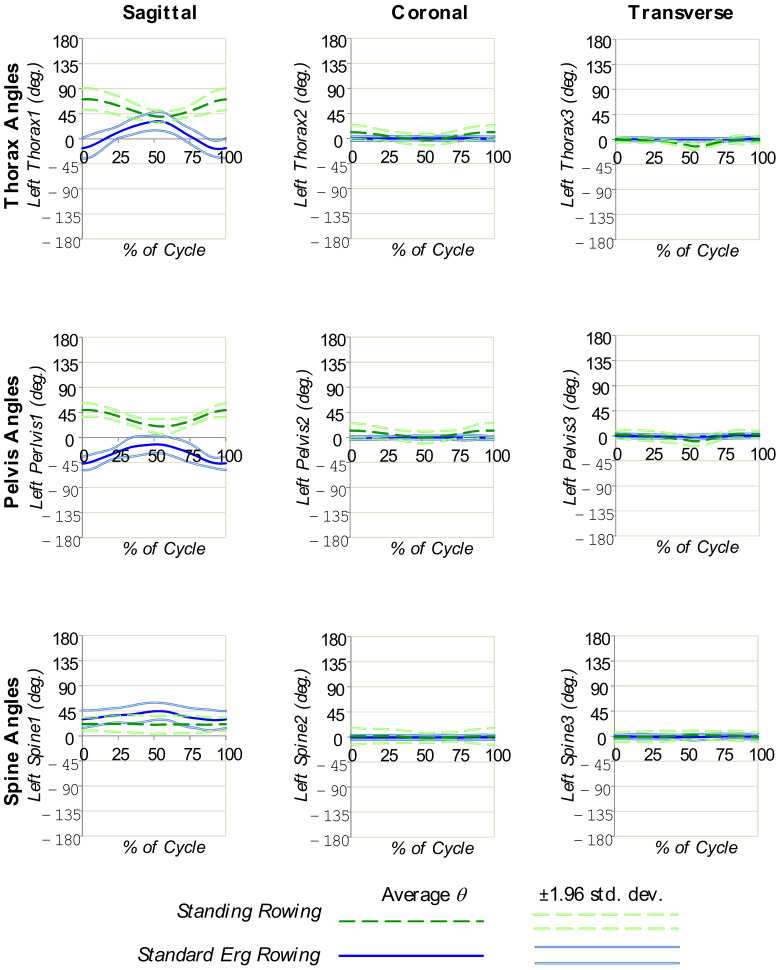
The averaged angular measurements for the thorax, pelvis and spine from the laboratory study, reported as mean ± 1.96 s.d., which compares standing rowing (green) with standard ergometer sliding-seat rowing (blue).

**Figure 6:**
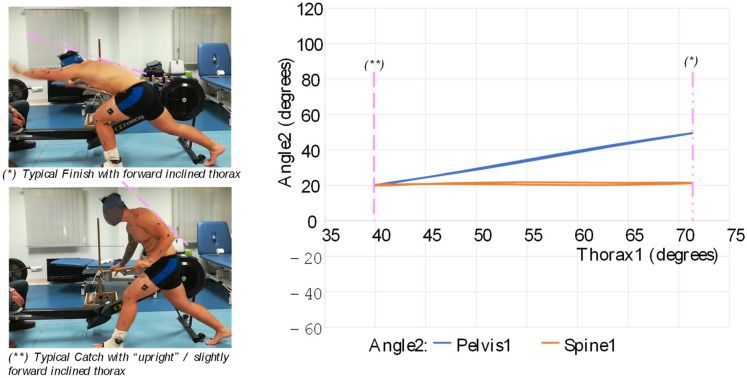
Angle–Angle plots, which illustrate the relationship between movements in sagittal plane of the pelvis/spine with that of the thorax. See also Supplementary Materials.

Measurements of the global orientation of the thorax, pelvis, and of the spine relative to the pelvis for standing rowing are reported in Figure 5, which provides a graphical report of how the measurements of the angular parameters vary, from finish to finish. These angular parameters are reported as averaged results ±1.96 standard deviations (where the value of ±1.96 should not be taken as implying that the data are normally distributed). This figure also compares this data with its equivalent related to standard ergometer rowing (the control group). To facilitate interpretation, Figure 6 shows a re-plot of the sagittal plots in Figure 5, in the format of angle–angle plots, where the pelvis and spine angular measurements are plotted against the thorax angles. A more complete set of the angle–angle graphs is shown in the Supplementary Materials. 

In addition, reported in Table 1, Table 2 and Table 3 are the ranges of motion *θ*_ROM_, the minimum and maximum values (*θ*_min_ and *θ*_max_) of each angular measurement plotted in Figure 5, together with the *p*-values related to a comparison of the average angular parameters. Note that only angles related to the left side of the body are being reported as the measurements of the angles in the sagittal, transverse, and coronal planes are related through symmetry between the left and right (equality for sagittal movements; mirror image for the transverse and coronal planes), as expected.

**Figure 7:**
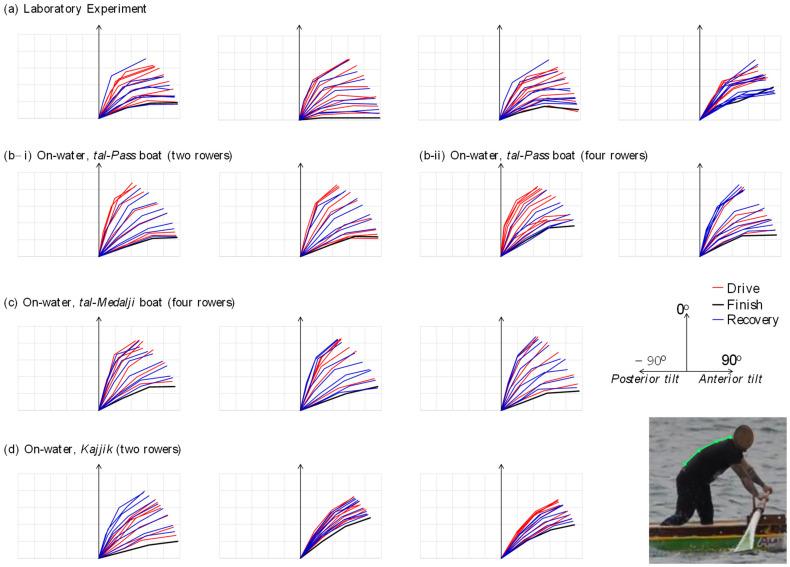
Images showing the typical outline of the back profiles traced from images extracted from video of on-water rowing and the lab study for standing rowing, where (**a**) shows cycles from each of the different rowers in the laboratory study, whilst (**b**–**d**) refer to the equivalent profiles from rowers rowing on different types of boats. The boats studied are all traditional Maltese rowing racing boats where (**b**) is the widest and heaviest *Tal-Pass* boat which can be rowed by two rowers, one sitting one standing (**b-i**), or four rowers, two sitting, two standing (**b-ii**); (**c**) refers to the more slender, slightly longer, and lighter *Tal-Medalji* version, also rowed by four rowers, two sitting, two standing whilst (**d**) refers to the much shorter *Kajjik*, rowed by two rowers, one sitting, one standing. The rower analysed is always the standing rower with the oar on his right-hand side (*parasija*). The manner of how these profiles were drawn is illustrated in the insert. Note that profiles refer to rowers facing the right direction.

Moreover, Figure 7 reports the traced outlines of the back profiles for various standing rowers whilst rowing on-water, which are meant to be treated as semi-quantitative estimates to support the quantitative results reported above. 

### 3.2. Measurements Related to Kinematics of the Knee, Ankle and Hip Joints

**Figure 8:**
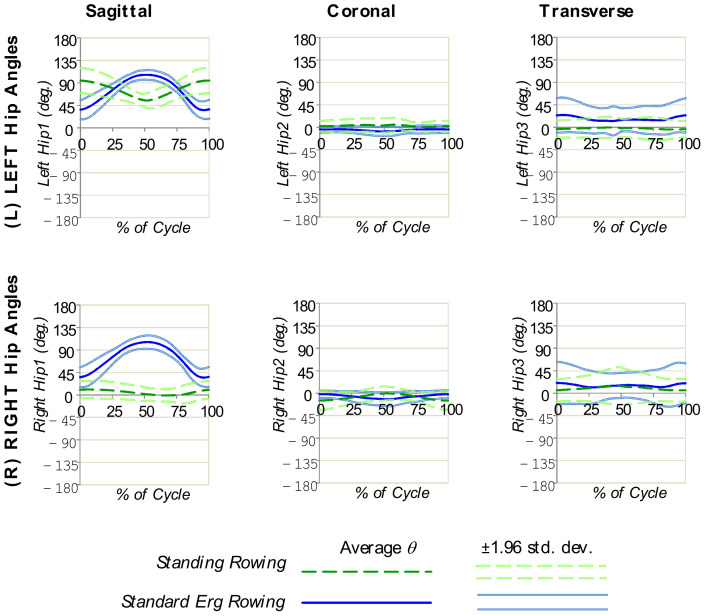
The averaged angular measurements for the hip from the laboratory study, reported as mean ± 1.96 s.d., which compares standing rowing (green) with standard ergometer sliding-seat rowing (blue).

**Figure 9:**
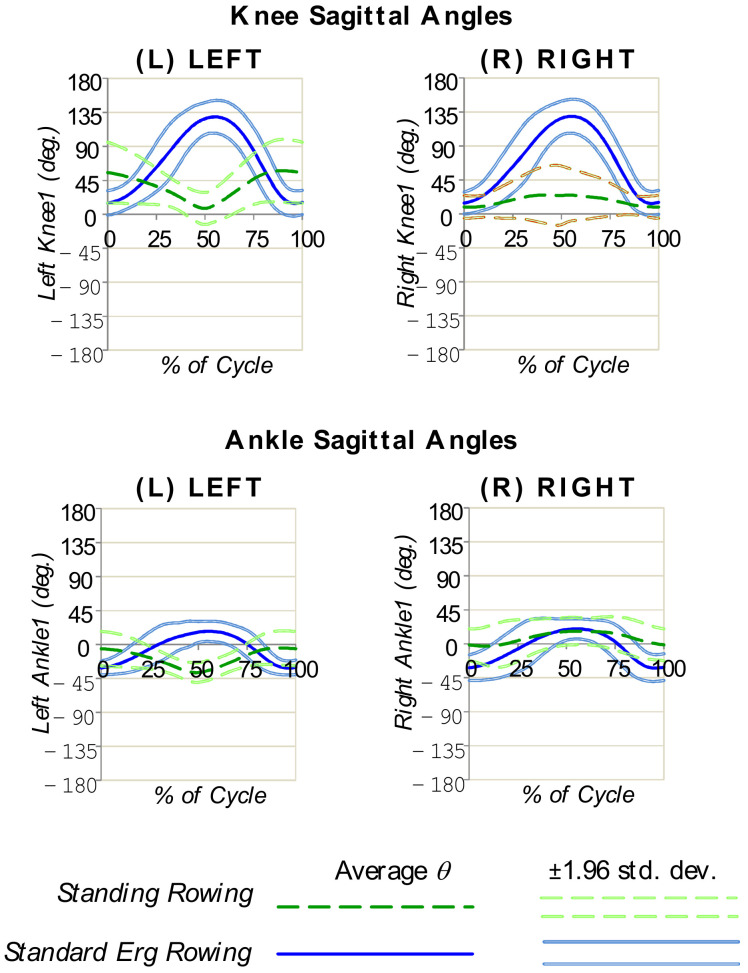
The averaged angular measurements for the knee and ankle (sagittal) from the laboratory study, reported as mean ± 1.96 s.d., which compares standing rowing (green) with standard ergometer sliding-seat rowing (blue).

**Figure 10:**
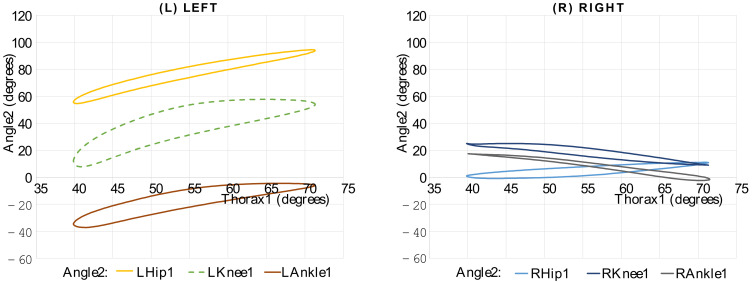
Angle–Angle plots which illustrate the relationship between movements in the lower body with that of the thorax. See also supplementary information for other plots.

Measurements related to the hip, knee and ankle are reported in Figure 8 and Figure 9. These plots report the variation, from finish to finish, of the angular parameters for the standing rowers as averaged results ±1.96 standard deviations, together with a comparison for standard ergometer rowing. For the sagittal plane, these data are also re-plotted, in the form of angle–angle plots, in Figure 10 to highlight the relationships between the various angular measurements and the inclination of the thorax. Moreover, Table 4, Table 5 and Table 6 also report *θ*_ROM_, *θ*_min_ and *θ*_max_ for each angular measurement plotted in Figure 8 and Figure 9, together with the corresponding *p*-values related to a comparison of standing rowing with standard ergometer sliding-seat rowing. 

### 3.3. Measurements concerning the Shoulders and Elbows

**Figure 11:**
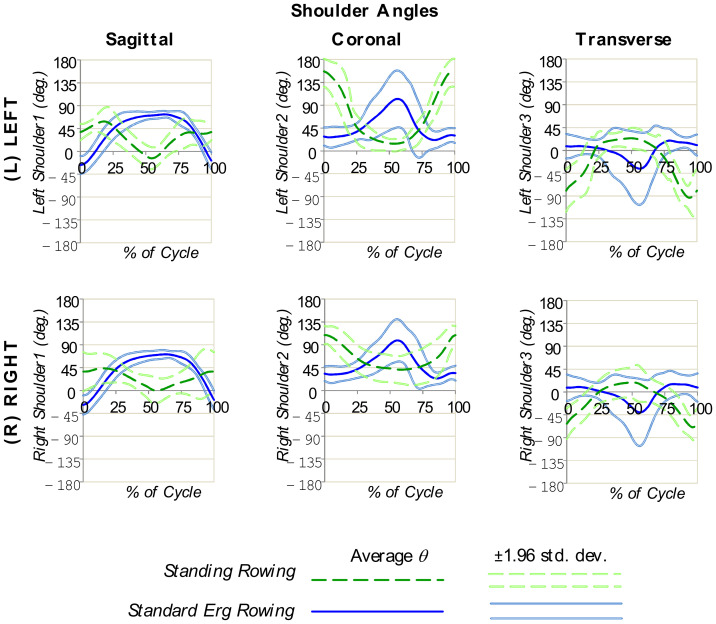
The averaged angular measurements for the shoulders from the laboratory study, reported as mean ± 1.96 s.d., which compares standing rowing (green) with standard ergometer sliding-seat rowing (blue).

**Figure 12:**
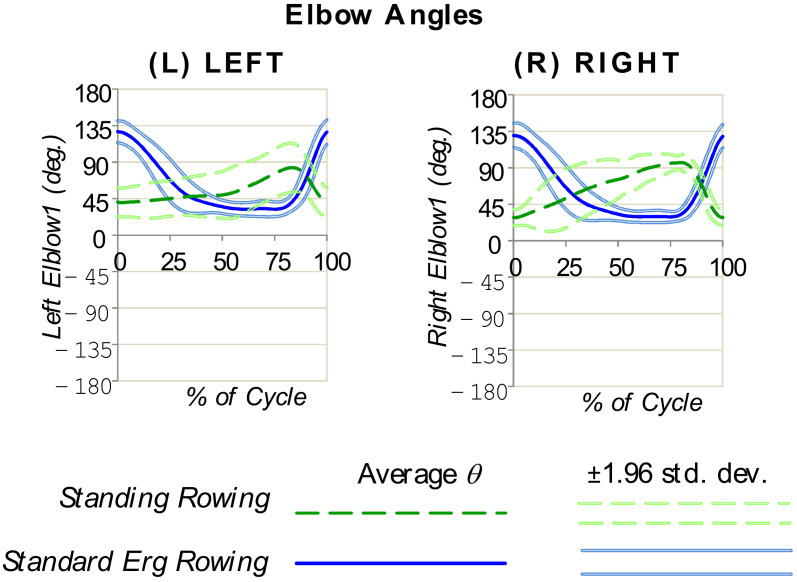
The averaged angular measurements for the elbow (sagittal) from the laboratory study, reported as mean ± 1.96 s.d., which compares standing rowing (green) with standard ergometer sliding-seat rowing (blue).

**Figure 13:**
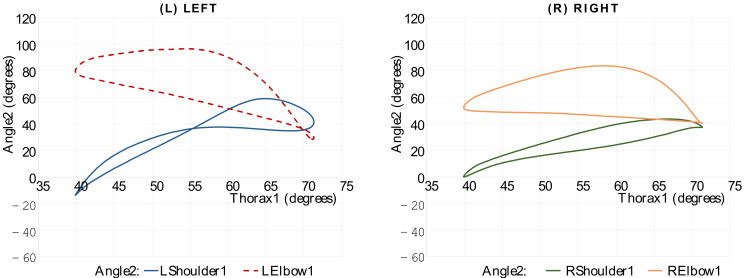
Angle–Angle plots which illustrate the relationship between movements in the shoulders and elbow with that of the thorax. See also Supplementary Materials for other plots.

Details of the averaged measurement results related to the shoulders and elbow, ±1.96 standard deviation from finish to finish, are reported in Figure 11 and Figure 12, respectively. For the sagittal plane, these data are also re-plotted, in the form of angle–angle plots, in Figure 13, to highlight the relationships between the various angular measurements and the inclination of the thorax. In addition, reported in Table 7, Table 8 and Table 9 are the corresponding values for *θ*_ROM_, *θ*_min_ and *θ*_max_ of each angular measurement plotted in Figure 9 and Figure 10, together with the corresponding *p*-values related to a comparison between standing rowing and standard ergometer sliding-seat rowing. 

## 4. Discussion

This discussion shall initially focus on the technique of standing rowers in the Venetian style, outlining the similarities to standard rowing and also aspects which are very different. 

From a practical aspect, an observation which deserves to be highlighted is that standing rowing is likely to be equally demanding as sitting rowing. Evidence for this claim comes from anecdotal evidence voiced by athletes who practise both forms of rowing and from mechanical analysis of the rigging used in Maltese boats. As noted in Figure 1, Figure 2 and Figure 3, oarlocks are generally placed in a *quasi*-perfectly anti-symmetric manner, practically equidistantly from the centre of the boat. With such rigging, since the oars used by the standing and sitting rowers are normally equal in length, the boat would only move in a straight direction as long as the standing and sitting rowers put the same amount of power on each respective side. This observation (which needs to be backed up by further studies using protocols typically used on Olympic boats [7,31,32,33,34,35,36]) is important since, in the absence of specific literature on standing rowing, one can, to a first approximation, hypothesise that findings from studies related to rowing in the standard form (e.g., studies on nutrition, physiology, etc.), are likely to be transferable to athletes who practise standing rowing. 

However, sitting and standing rowing differ from each other in a number of other aspects, including very important differences related to movements. In order to expand on the specificities of standing rowing, one would need to highlight what constitutes rowing which, such as walking, running, canoeing and several other sports, can be considered as a cyclic form of movement with numerous repetitions of the same motion. When practised as a sport, in the traditional classical form, the rower sits on a fixed seat and moves the boat primarily by means of the force generated by the trunk and arms with what can be considered as a “pull action” in the “drive” active highly endergonic phase in which the athlete expends energy to move the boat by bringing the oars towards her/himself. This is followed by a much less endergonic “recovery” which sees the athlete reverse back, with the oar outside the water for another active drive phase. This classical form of rowing is normally practised on traditional boats (e.g., the Cornish Pilot gigs, the Italian *Gozzo* or *Lancia*, the Spanish *trainera* or the Maltese “*Dgħajsa tal-Pass*” shown in Figure 1, Figure 2 and Figure 3), which are wider and heavier than their Olympic counterparts.

With time, the classic form evolved and in Olympic-type rowing, the athlete sits on a mobile seat (an invention which dates to the second part of the 19th century), and consequently the boat is moved with the use of the leg, trunk and arms, with the powerful lower limbs providing most of the power required to propel the boat through a “push action”. The “drive” phase in Olympic style rowing initiates at the catch when the blade enters the water with the athlete pushing with their lower limbs whilst keeping the arms straight and the trunk slightly inclined forwards. This is followed by “pulling” actions in the form of tilting the trunk posteriorly (from an “one-o-clock” position to an “eleven-o-clock” position), and finishing with a pull of the arms to bring the oar handles close to the chest and immediately expelling the oar/s out of the water and feather (the “finish” position). Once again, this highly endergonic phase is followed by the “recovery” phase, which essentially sees the athlete reversing from the “finish” position to the “catch” in preparation for the next cycle. 

Standing rowing, although practised on traditional boats, differs from the classic fixed-seat seated rowing as it involves a “pushing” action in the endergonic drive phase, rather than pulling. In addition, in contrast with standard sliding-seat rowing, the rower makes extensive use of the upper body in the “pushing action.” Such differences are graphically illustrated in Figure 1b and Figure 3 where one can observe the complimentary yet mechanically opposite actions of the seated and standing rowers. These opposing movements are also very evident through the plots of measurements taken in the sagittal plane, i.e., the plane where most movements are recorded, with standing and sitting measurements almost mirroring each other. Thus, for example, the trunk of the standing row would be leaning forwards whilst that of the seated rower is leaning backwards (see Figure 5) or the elbows of the standing rower are in extension whilst those of the seated rower flexed (see Figure 10). As a result, one could argue that from the perspective of a workout, these two forms of rowing—seating and standing—provide the athlete with a highly complementary set of movements, which if used together could be highly beneficial for the development of the athletes. Moreover, from the perspective of a physical exercise, it is important to highlight that standing rowing, with its demands for stability and balance in the boat (the athlete is standing on a moving and somewhat unstable shell), provides an excellent complimentary exercise to the standard isometric and dynamic balance exercises for the physical conditioning of the athlete.

Keeping these factors in mind, the discussion will now focus on the specific kinematics of standing rowing in the Venetian style, aiming at a better understanding of this technique, enabling replication and possibly further development. 

### 4.1. The Thorax, Pelvis and Spine Kinematics of Standing Rowers

The analysis of the kinematics of standing rowers in the Venetian style is best commenced through the presentation of a selection of consecutive frames within a rowing cycle, such as the ones shown in Figure 3 and Figure 4, focusing on back profiles, which are plotted in Figure 6. These images allow for a very visual appreciation of the main movements associated with this form of rowing, i.e., one where the rower moves from a quasi-standing but inclined forward position at the catch (which corresponds to the sagittal thoracic angle Thorax1 being at *θ*_min,_ averaging around 40°) to one where the rower, sometimes, has the back parallel to the water surface or the laboratory floor towards the finish (which corresponds to Thorax1 being at *θ*_max_, averaged around 72°, but reaching as much as 83° in the case of Rower 2). This latter highly forward flexed position of the thorax is held for quite some time during the rowing cycle and provides two important advantages: (1) It elongates the reach at the finish permitting for a larger arc of travel for the blade that results in forward propulsion (2) the centre of gravity of the rower is lowered, stabilising both himself and the boat. It is interesting to note that the range of motion of the thorax for the standing rowers averages 32°. Here, it must be said that, from the data collected, as well as from actual observation of Venetian style rowing practised on water, the range of motion, as well as extent of forward leaning, seems to be a personal preference of the rowers. Our laboratory study, which looked at four rowers, analysing three cycles per rower, observed one rower who had a range of motion of c. 18°, indicating that the rower performs very little motion forwards and backwards, whilst another had a range of motion of 48° indicating a substantial forward and backwards motion. Irrespective of personal preference, this movement is generally significantly different (lower) to that of the seated rowers, to the extent that the *p*-value for a comparison between the mean *θ*_ROM_ for Thorax1 standing vs. standard ergometer rowing is c. 0. 

The pelvic movement in the sagittal plane follows a similar trend with the recorded range of motion indicating a synchronised movement between the pelvis and the thorax, with the result that spinal movement is rather low, averaging at 7°. This clearly suggests that the rowers are keeping their lumbar spine erect during the motion with most of the movement occurring at the pelvis (or rather the hip as the pelvis can only attain a small degree of anteversion and retroversion [37]. 

When viewing the coronal and transverse angular results, it is notable that in contrast to standard ergometer rowing, there is considerable sideways tilting and rotational twisting, with *θ*_ROM_ averaging at c. 15° for both Thorax2 (coronal) and Thorax3 (transverse). In the case of coronal movements, maximum sideways tilting occurred at the finish. Once again, this indicates that to arrive at the finish with maximum drive length, the trunk exhibits a degree of lateral motion to starboard (in the case of the *Parasija*), with the maximum angle reached by one of the rowers being c. 17° (which was not insignificant). This high range of motion associated with thoracic movements in the coronal plane seems to be a characteristic feature of standing rowing, something which differs significantly from standard sliding-seat erg rowing (*p*-value < 0.0005). The extent of thoracic transverse plane rotations, Thorax3, is also significantly higher to that observed in standard ergometer rowing, but such rotation is a known feature of sweep rowing as practised by seated rowers. In fact, this rotational component shows that rowers are in a twisted form at the catch and straighten at the finish. This change in posture to assume a non-rotated trunk at the finish has the advantage of a longer and firmer push when reaching the finish point whilst the rather large transverse plane angles at the catch result in an elongation of the drive length. 

At this point, it is interesting to make remarks with regard to the individuality shown by the rowers, very evident through the comparison of their thorax. Casual observation of various races and training sessions seems to suggest that (i) different standing rowers adopt different postures and nuances of technique; (ii) rowers may choose to alter their posture during a race, which is generally by design as standing rowers take the role of steering the boat, something which they do by varying slightly their technique to either focus on pushing forward straight, or implementing some directional changes.

### 4.2. Knee, Ankle and Hip Kinematics of Standing Rowers

One of the most prominent features of standing rowing is that the left and right knees have very asymmetrical movements. In fact, for the *Parasija* position, the rower stands with his feet in a walk-standing posture where the left lower limb leads the movement and moves significantly in the rowing cycle. The range of motion of the left knee averaged at 52° (s.d. 13.4°) with a rather wide range from c. 38° to c. 65°. The catch (which roughly corresponds to the left knee at *θ*_min_) and finish angles which roughly correspond to the left knee at *θ*_max_ were also very individualised, with one rower starting with catch angles as low as c. −3° (i.e., in hyperextension) and other starting at c. 23°, whilst the finish angles were sometimes as large as c. 89° (meaning an almost perpendicular knee stance) to less than 40°. This limb serves to pivot the body forwards as the rower pushes on the oar. It also provides directional stability during the rowing cycle and involves hip and ankle movements to perform the optimal cycle. This variation may be due to several reasons, such as anthropometric features, and personal preferences such as design, seeking more comfort/stability. 

The right knee demonstrates very different behaviour and it seems that some rowers prefer to keep their knee quasi-static throughout the cycle (Rowers 1 and 4) whilst others maintain a flexed position (Rowers 2 and 3). In addition, the lab results suggest that the rowers that do not flex their right knee, tend to assume different positions ranging from a quasi-straight leg during the whole cycle (as was the case with Rower 4) whilst others keep this knee slight bent (as was the case with Rower 1). Nevertheless, in all cases, movements in the left side (for the *Parasija*) are of larger degrees than on the left with visual analysis of on-water rowing confirming that in on-water rowing, some standing rowers tend to flex their left knee to c. 90°. 

Here, it must be re-emphasised that there is a noticeable difference in function between the left and right lower limbs in standing rowing. The right hip serves as the stance leg in that it is posterior to left and pivots mainly on the metatarsal heads of the foot. The right ankle is held in a particular position during standing rowing. Standing rowers all exhibit range of motion changes in the left ankle which is explained by the ankle being in a relative plantar flexed position (around 1°) at the catch due to the shank being in extension, in line with the rest of the lower limb. This then changes into a dorsiflexed position of around 16.5° maximum at the end of the drive phase. Here, it must be noted that the pressure through the ball of the foot through the posterior support plate enhances the overflow of energy and stimulation to contract in the muscles of the right lower limb, a principle known as ‘overflow’ after Sherrington (1906) [38].

When analysing nuances of the technique of the four standing rowers, it was only Rower 4 who demonstrated barely any movement of the hip, which could be due to his shorter stature and higher BMI and the fact that his technique was remarkably different to the other three rowers. In addition, Rower 1 and Rower 3 were shown to abduct the hip at a certain phase of the cycle whilst Rower 4 maintained lateral rotation throughout the cycle. Once again, through observation, Rower 4 exhibited a rather different movement pattern of the left lower limb, where, besides the laterally rotated hip, the tibia also rocked into lateral rotation and the knee travelled into hyperextension. This pattern was completed with inversion of the foot at the subtalar joint.

Referring to views in the coronal plane, an interesting point was that the taller two of the four rowers (Rower 1 and 3) showed more lateral movement than the other two. This may have the possible implication that taller rowers show more lateral movements; however, one has to keep in mind that techniques vary greatly from one rower to the next. 

### 4.3. The Shoulder and Elbow Kinematics of Standing Rowers

The profile of the angles associated with shoulder movements are unique for standing rowing. For example, a striking feature is that the peaks of the angles in the sagittal plane were not reached at the catch or finish, something that is generally the case with seating rowing, where the catch and finish represent extreme positions. Instead, for the left shoulder, where the arc of shoulder movements is most pronounced for the *Parasija* position, very distinct peak sagittal shoulder angles are encountered twice in cycle: once in mid-recovery (the recovery is initiated with coronal and transverse plane movements which are the most dominant until mid-recovery and the sagittal plane motion only becomes the predominant one after mid recovery), during mid-drive. This double peaking suggests that the shoulder complex plays a very prominent role in standing rowing and the complex and fast movements have to be executed in anatomically and biomechanically correct patterns to produce optimal movements which maximise the length of the drive and its power, in a comfortable yet efficient manner. In the case of the right shoulder, there is some similar, but less pronounced sagittal peaking in mid-recovery, as expected, since both arms must move in synchrony as they hold the same oar. One may observe that peaking in the transverse plane occurs mid-drive, a movement which is necessary due to lateral scapular rotation which allows the glenohumeral joint to rotate superiorly, thus allowing elevation of the humerus with a clear subacromial space. From a performance perspective, this movement at the final part of the drive is essential to elongate the reach at the finish by increasing the arc of the oar blade. 

As evidenced from the images depicting standing rowing, there are highly pronounced shoulder movements in standing rowing as the rower uses his shoulder muscles to push, rotate, and stabilise the oar. Once again, apart from discussing the average behaviour, recognising that each rower is different and thus typically performs the rowing action in a somewhat personalised manner, it is also important to discuss any individual variations that one may observe. This personal variation is highly evident in shoulder kinematics, where the data suggest a rather wide range of shoulder angles when comparing the different rowers. However, in general, the angles in the sagittal plane for the left shoulder are at a minimum near the catch. This is explained by the fact that, for standing rowing, the catch commences at a point where the oar is close to the body of the rower as opposed to seated rowing. Thus, the shoulders are kept in a position of extension with the range, once again depending on the anthropometric and rowing-style attributes of the individual rower. At the finish point, the sagittal shoulder angles average to c. 37–38°, with quite a bit of variation between rowers, especially in the left shoulder measurements. This somewhat anomalous value is, when one views the actual action, a dip of the glenohumeral joint towards a slight extension at the finish, which shows the arm in *quasi-*full elevation. However, the fact that the trunk is flexed may explain the interpretation of extension at the finish point. This may possibly be due to the position of the subject in the capture envelope of the motion capture cameras where the angles of the markers are calculated relative to a plane traversing the neck and lower down the pelvis. Hence, with the arm traversing this imaginary plane, the software translates it into an extension movement. However, it must be emphasised that maximum shoulder angles are not demonstrated neither at the catch nor at the finish, but halfway through the recovery as the rower is pushing back the oar to its original catch position. It must also be said that the profile of the blade in the water for standing rowing is much more circular than oval (as is the case of sitting rowing), thus explaining the wide range of motion of the shoulder angles. The coronal movement also reveals differences between the left and right shoulder angles where the left goes through a larger arc of abduction compared to the right. 

In the case of the elbow movements for standing rowing, once again, the most interesting feature is that the peak movement is occurring mid-drive and not at the catch or finish. This movement corresponds to the final push that rowers give to the oars just prior to the finish, which in the case of seated rowers is accomplished by feathering in the water, and which gives the heavy boats the impulse required to keep their speed during the recovery. 

### 4.4. Strengths and Limitations of This Work

This work investigated the biomechanics and kinematics of Venetian style standing rowing and compares it with what is known about the standard and traditional seated forms of rowing. The main strength of this work is that this work reports the kinematics of a technique that has never, to the authors’ knowledge, been studied quantitatively and documented for other to replicate and further develop. In doing so, this work identified some aspects that were either previously ignored or never formally recorded or studied. These include the fact that for the first time, it is acknowledged that Venetian-type rowing may be as efficient and effective as its seated counterpart, and in many aspects complementary to it. All this will hopefully permit better recognition of this most elegant rowing technique, and possibly also encourage others to try it. From a very practical aspect, it should be noted that being forward-facing, standing rowing could be particularly useful to help one navigate and steer a boat in busy stretches of water. 

An important strength is that methodology adopted, which examines the rowers in a state-of-the-art calibrated laboratory setting rather than on-water, eliminates the limitations associated with taking on-water measurements. However, the use of on-water data to compliment the laboratory findings gives peace of mind that what is being reported here is likely to be a true replica of the real on-water scenario. Moreover, this work has shown how a standard sliding-seat rowing ergometer can be further developed to emulate standing rowing, something which is likely to be appealing to athletes, coaches and gym users as a novel and effective training tool for physical conditioning. Recognising that the sports industry is known to be constantly on the lookout for new ideas for developing new tools and devices for physical conditioning and a complete workout that replicates the sports movement (e.g., rowing ergometer, canoe ergometer, the ski-erg, etc.), it is hoped that this concept will be further developed and ultimately become mainstream. 

Limitations include the fact that with work focused purely on kinematic aspects in a highly idealistic laboratory scenario which, for example, did not have into account the instability provided by the water, which might influence motor control and kinematic parameters. Another limitation of this work was that the rowers were permitted to row at their own pace. Whilst this had the advantage that rowers felt at ease during the experiment, it had the disadvantage that the data generated needed to be processed in order to make the catch and finish points coincide (a simple process where the average occurrence of the “catch” within the “finish to finish” cycles was identified and the length of the drive and recovery phases appropriately scaled so that “finish” and “catch” are synchronised). Moreover, the number of subjects studied was small and no attempt was made to standardise the data (apart from reporting the cycle as a percentage and aligning the ‘catch’ and ‘finish’). Given that it is well known that kinematics is highly affected by height, span, etc., it would be useful if such factors are taken into consideration in further studies. Moreover, it would have been ideal to link technique to results obtained in races, something which was not performed for ethical reasons. Moreover, no attempt was made to study how standing rowing could physically affect the athletes practising it (e.g., changes in body composition, effect on strength, muscle mass, tendons, bone strength, etc. [39,40]), or how athletes alter their technique when they have muscle tightness [30], become fatigued or blistered [26], or if they need to augment or decrease the power to increase boat speed or to steer the boat. Ideally this study should be extended to look into these issues, measure muscle activity, forces, etc.

Another important limitation is that this work only looked at rowers who hold the oar on their left-hand side (the “parasija” position). Whilst there are no reasons to suggest that the standing rowers on the other board would row differently, apart from rowing in “mirror image”, it would be useful if in the future the kinematics of rowers on the opposite board are also studied. 

Finally, an important limitation is that this work only looked at Venetian-style standing rowing through the manner how this is practised as a sport in Malta. Whilst this had the advantage that in Malta, standing rowers row on the same boat with the same stroke as sitting rowers, it would have been useful to also examine rowers who hail from different countries to assess if the technique practised in Malta differs in any way from that practised in other countries. 

## 5. Conclusions

This work has for the first time,

- Documented the kinematics of standing Venetian style rowing, as performed by abled-bodied athletes through a laboratory-based study supplemented by data recorded on-water in a manner which permits this historical form of rowing made famous by the gondoliers of Venice to be replicated by others and form the basis for further studies and developments;
- Measured and reported the range of motion of various joints, thus showing that it constitutes a whole-body exercise which is highly complementary to standard and traditional seated rowing;
- Highlighted some unique advantages associated with this form of rowing, such as that it can be performed by an athlete facing forward rather than backwards, thus possibly making it safer to practise in busy waters.

Hopefully, with this first ever toolkit to help athletes and coaches clearly understand how competitive Venetian-style standing rowing is practised, this form of rowing will grow in popularity through a wider uptake by new athletes and further studies. In particular, further studies could look into muscle activity, energetics, injuries, etc., and also look into the possibility of making this form of rowing accessible to individuals with impairments, in an analogous way that para-rowing exists alongside Olympic rowing, something which so far seems to have never been attempted for Venetian-style rowing.

## Figures and Tables

**Table 1:** The range of motion values, *θ*_ROM_, of the thorax, pelvis and spine angles measured for the Venetian style rowing technique, in degrees, ± 1 s.d., for the individual rowers 1–4, their mean, and its equivalent mean value for standard ergometer rowing, with *p*-value for these two means reported in parentheses.

*θ*_ROM_		Venetian Rowing Technique (VRT)					Control–Erg	
		Rower 1	Rower 2	Rower 3	Rower 4	Mean	Mean	(*p*)
Thorax1	L	24.7 ± 3.1	47.7 ± 1.2	39 ± 4.4	18 ± 3	32.3 ± 12.5	52 ± 11.3	(0.000)
Thorax2	L	12 ± 1	17 ± 1	21.3 ± 2.5	9.3 ± 2.9	14.9 ± 5.1	2.9 ± 1.2	(0.000)
Thorax3	L	10.7 ± 1.5	11.7 ± 0.6	20.3 ± 4.2	15.3 ± 2.9	14.5 ± 4.6	3.3 ± 1.6	(0.000)
Pelvis1	L	29.3 ± 2.9	44 ± 2	30.3 ± 1.5	18 ± 5.6	30.4 ± 10	36.3 ± 9.3	(0.139)
Pelvis2	L	12 ± 1.7	10 ± 2	21.7 ± 1.5	9.7 ± 2.1	13.3 ± 5.3	2.7 ± 1.5	(0.000)
Pelvis3	L	10.3 ± 2.1	13 ± 1	20.7 ± 3.8	15 ± 3.6	14.8 ± 4.7	3.5 ± 1.6	(0.000)
Spine1	L	9.3 ± 1.5	7.3 ± 1.5	7.3 ± 2.1	4 ± 1	7 ± 2.4	17.3 ± 6.6	(0.000)
Spine2	L	6.3 ± 0.6	5.7 ± 1.5	6.7 ± 1.2	5.7 ± 2.1	6.1 ± 1.3	2.8 ± 0.9	(0.000)
Spine3	L	7.3 ± 1.5	5.3 ± 1.5	4.7 ± 1.2	4.7 ± 1.2	5.5 ± 1.6	2.9 ± 1	(0.000)

**Table 2:** The minimum values, *θ*_min_, of the thorax, pelvis and spine angles measured for the Venetian style rowing technique, in degrees, ± 1 s.d., for the individual rowers 1–4, their mean, and its equivalent mean value for standard ergometer rowing, with *p*-value for these two means reported in parentheses.

*θ*_min_		Venetian Rowing Technique (VRT)					Control–Erg	
		Rower 1	Rower 2	Rower 3	Rower 4	Mean	Mean	(*p*)
Thorax1	L	46.7 ± 3.2	35 ± 1.7	37 ± 2.6	39.3 ± 2.1	39.5 ± 5.1	−20 ± 7.6	(0.000)
Thorax2	L	3.3 ± 1.5	0 ± 1	−7.3 ± 2.5	−7.3 ± 1.5	−2.8 ± 5.1	−1.4 ± 2.2	(0.427)
Thorax3	L	−11.3 ± 0.6	−12 ± 1.7	−16.3 ± 3.5	−14 ± 1.7	−13.4 ± 2.7	−1.5 ± 1.3	(0.000)
Pelvis1	L	15.7 ± 2.5	11.7 ± 3.2	24.3 ± 1.5	26 ± 5.3	19.4 ± 6.9	−48.3 ± 4.7	(0.000)
Pelvis2	L	7.3 ± 1.2	−4.3 ± 1.2	−3.7 ± 0.6	−3.3 ± 2.5	−1 ± 5.2	−2.1 ± 1.6	(0.000)
Pelvis3	L	−8.3 ± 1.2	−8.7 ± 0.6	−17.7 ± 3.5	−7 ± 1	−10.4 ± 4.7	−2.8 ± 1.7	(0.000)
Spine1	L	24.3 ± 1.2	21.3 ± 2.5	13.3 ± 1.2	11.3 ± 2.5	17.6 ± 5.9	27.4 ± 8.6	(0.002)
Spine2	L	−0.3 ± 2.1	−11.3 ± 2.9	−0.7 ± 1.5	0.7 ± 2.5	−2.9 ± 5.5	−2.3 ± 1.9	(0.397)
Spine3	L	−6.3 ± 2.3	−2.7 ± 0.6	−4.7 ± 0.6	4 ± 1	−2.4 ± 4.3	−2.6 ± 2.6	(0.577)

**Table 3:** The maximum values, *θ*_max_, of the thorax, pelvis and spine angles measured for the Venetian style rowing technique, in degrees, ± 1 s.d., compared to its equivalent mean value for standard ergometer rowing.

*θ*_max_		Venetian Rowing Technique (VRT)					Control–Erg	
		Rower 1	Rower 2	Rower 3	Rower 4	Mean	Mean	(*p*)
Thorax1	L	71 ± 1	82.7 ± 1.5	76 ± 2	57 ± 3.5	71.7 ± 10	32.2 ± 8.5	(0.000)
Thorax2	L	15.3 ± 2.5	16.7 ± 0.6	14.3 ± 0.6	2 ± 2	12.1 ± 6.3	1.7 ± 1.8	(0.000)
Thorax3	L	−0.3 ± 0.6	−0.3 ± 1.2	4 ± 1	1.7 ± 2.5	1.3 ± 2.3	1.8 ± 1.7	(0.314)
Pelvis1	L	45 ± 1.7	55.3 ± 4.7	55 ± 1	43.7 ± 0.6	49.8 ± 6.1	−12 ± 8.3	(0.000)
Pelvis2	L	19.7 ± 2.1	5.3 ± 2.1	18 ± 2	6.7 ± 0.6	12.4 ± 6.9	0.8 ± 2	(0.000)
Pelvis3	L	2 ± 3	4.3 ± 1.5	3 ± 1.7	8.3 ± 4	4.4 ± 3.4	0.7 ± 2	(0.001)
Spine1	L	33.7 ± 2.1	29 ± 2.6	20.3 ± 2.5	15 ± 3.6	24.5 ± 8	44.8 ± 7.8	(0.000)
Spine2	L	6 ± 1.7	−6.3 ± 1.2	5.7 ± 2.1	6.3 ± 1.5	2.9 ± 5.8	0.4 ± 2.2	(0.015)
Spine3	L	0.3 ± 1.2	2.7 ± 1.5	0 ± 1	9 ± 1	3 ± 3.9	0.3 ± 2.6	(0.061)

**Table 4:** The range of motion values, *θ*_ROM_, of the hip, knee and ankle angles measured for the Venetian style rowing technique, in degrees, ± 1 s.d., for the individual rowers 1–4, their mean, and its equivalent mean value for standard ergometer rowing, with *p*-value for these two means reported in parentheses.

*θ*_ROM_		Venetian Rowing Technique (VRT)					Control–Erg	
		Rower 1	Rower 2	Rower 3	Rower 4	Mean	Mean	(*p*)
Hip1	L	43.7 ± 2.5	49.3 ± 4.7	48 ± 1	23 ± 3.6	41 ± 11.4	72.5 ± 9.9	(0.000)
	R	16.3 ± 1.5	23 ± 2.6	6.3 ± 2.1	9 ± 1	13.7 ± 7	72.4 ± 10.2	(0.000)
Hip2	L	8.7 ± 0.6	3.3 ± 0.6	7.3 ± 2.1	5 ± 1	6.1 ± 2.4	7.7 ± 3.4	(0.236)
	R	14 ± 1.7	9.3 ± 2.5	25.7 ± 2.1	12.7 ± 3.2	15.4 ± 6.8	11.2 ± 5.2	(0.162)
Hip3	L	7 ± 1	9.7 ± 1.5	4 ± 1.7	9.7 ± 1.2	7.6 ± 2.7	16.9 ± 6.5	(0.000)
	R	18 ± 8	4.7 ± 1.2	7.3 ± 2.1	17 ± 15.6	11.8 ± 9.7	18.5 ± 5.8	(0.008)
Knee1	L	65.3 ± 1.5	37.7 ± 3.2	64.3 ± 3.1	42.3 ± 4.5	52.4 ± 13.4	116.6 ±13.1	(0.000)
	R	11.3 ± 2.5	37.7 ± 4.2	30 ± 0	11 ± 3.6	22.5 ± 12.4	116.8 ±14.6	(0.000)
Ankle1	L	37.7 ± 0.6	28.3 ± 2.5	45 ± 1	26 ± 4.4	34.3 ± 8.2	49.4 ± 5.8	(0.000)
	R	36 ± 3.5	19.7 ± 1.5	14.3 ± 4.2	21.7 ± 5.1	22.9 ± 9	52.7 ± 6.3	(0.000)

**Table 5:** The minimum values, *θ*_min_, of the hip, knee and ankle angles measured for the Venetian style rowing technique, in degrees, ± 1 s.d., for the individual rowers 1–4, their mean, and its equivalent mean value for standard ergometer rowing, with *p*-value for these two means reported in parentheses.

*θ*_min_		Venetian Rowing Technique (VRT)					Control–Erg	
		Rower 1	Rower 2	Rower 3	Rower 4	Mean	Mean	(*p*)
Hip1	L	61.7 ± 3.2	42.7 ± 2.5	57.7 ± 1.2	52.7 ± 2.9	53.7 ± 7.7	34.5 ± 8.7	(0.000)
	R	−11.7 ± 1.2	2 ± 1	0.3 ± 0.6	0.3 ± 2.5	−2.3 ± 5.9	33.9 ± 9.2	(0.000)
Hip2	L	7.3 ± 2.1	−3 ± 1	−1.7 ± 1.2	−3.7 ± 3.2	−0.3 ± 5	−9.6 ± 5	(0.000)
	R	−23.3 ± 1.5	−9.7 ± 2.3	−24.3 ± 1.5	−3.7 ± 0.6	−15.3 ± 9.3	−12.5 ± 7.2	(0.346)
Hip3	L	4.3 ± 1.2	−10 ± 1	−18.7 ± 1.5	−1 ± 1	−6.3 ± 9.2	9.8 ± 15.5	(0.004)
	R	10 ± 1	−13.7 ± 1.2	5.3 ± 2.9	16 ± 1.7	4.4 ± 11.7	6.8 ± 17.4	(0.401)
Knee1	L	23 ± 1.7	4.7 ± 0.6	1.3 ± 1.5	−3.3 ± 1.5	6.4 ± 10.5	13.1 ± 8.4	(0.025)
	R	13.7 ± 1.5	13 ± 1	3.7 ± 0.6	−3.7 ± 8.6	6.7 ± 8.4	12.9 ± 7.8	(0.055)
Ankle1	L	−28 ± 2	−43.7 ± 2.3	−41.3 ± 1.2	−40 ± 4.6	−38.3 ± 6.8	−32 ± 4.4	(0.006)
	R	−18.3 ± 5.1	7 ± 1.7	8.7 ± 1.2	−16.7 ± 2.1	−4.8 ± 13.5	−33 ± 9.5	(0.000)

**Table 6:** The maximum values, *θ*_max_, of the hip, knee and ankle angles measured for the Venetian style rowing technique, in degrees, ± 1 s.d., for the individual rowers 1–4, their mean, and its equivalent mean value for standard ergometer rowing, with *p*-value for these two means reported in parentheses.

*θ*_max_		Venetian Rowing Technique (VRT)					Control–Erg	
		Rower 1	Rower 2	Rower 3	Rower 4	Mean	Mean	(*p*)
Hip1	L	105.3 ± 1.5	91.7 ± 6.8	105.7 ± 0.6	76 ± 1	94.7 ± 13.1	106.8 ± 4.8	(0.005)
	R	4.7 ± 0.6	25 ± 2.6	6.3 ± 2.1	9.7 ± 3.1	11.4 ± 8.6	106.3 ± 6.9	(0.000)
Hip2	L	16 ± 2	0.7 ± 1.5	5.3 ± 1.2	1 ± 2	5.8 ± 6.6	−2 ± 3	(0.000)
	R	−9 ± 1	−0.3 ± 3.2	1.3 ± 1.2	9.3 ± 2.9	0.3 ± 7.1	−1.4 ± 4.1	(0.000)
Hip3	L	11 ± 2	−0.3 ± 1.5	−15 ± 1	8.7 ± 1.5	1.1 ± 10.7	26.8 ± 16.3	(0.000)
	R	28 ± 8.2	−8.7 ± 0.6	12.3 ± 2.3	33 ± 14	16.2 ± 18.3	25.4 ± 15.9	(0.000)
Knee1	L	88.7 ± 2.9	42 ± 3.6	65.3 ± 1.5	39 ± 5.6	58.8 ± 21.2	129.7 ± 11	(0.000)
	R	24.7 ± 1.5	50 ± 4.6	33.7 ± 0.6	7.3 ± 7.8	28.9 ± 16.6	129.7 ±11.4	(0.000)
Ankle1	L	9.7 ± 1.5	−15.3 ± 1.5	3.7 ± 0.6	−13.7 ± 3.8	−3.9 ± 11.4	17.5 ± 6.8	(0.000)
	R	17.7 ± 3.5	27 ± 1	23 ± 3.6	6 ± 4.4	18.4 ± 8.7	19.9 ± 7	(0.000)

**Table 7:** The range of motion values, *θ*_ROM_, of the shoulder and elbow angles measured for the Venetian style rowing technique, in degrees, ± 1 s.d., for the individual rowers 1–4, their mean, and its equivalent mean value for standard ergometer rowing, with *p*-value for these two means reported in parentheses.

*θ*_ROM_		Venetian Rowing Technique (VRT)					Control–Erg	
		Rower 1	Rower 2	Rower 3	Rower 4	Mean	Mean	(*p*)
Shoulder1	L	64.3 ± 5.9	79.3 ± 2.1	80.3 ± 4.2	84 ± 3.6	77 ± 8.6	101.5 ± 7.9	(0.000)
	R	22.7 ± 3.1	54.7 ± 3.1	35 ± 6.9	85.3 ± 3.1	49.4 ± 25	100.6 ± 9.7	(0.000)
Shoulder2	L	152 ± 3.5	157.7 ± 2.5	162.3 ± 4.7	108 ± 5.3	145 ± 22.9	86 ± 27.7	(0.000)
	R	44.7 ± 7.5	85.7 ± 8.7	75.3 ± 3.2	83.3 ± 11	72.3 ± 18.5	80.2 ± 21.8	(0.591)
Shoulder3	L	137.7 ± 3.8	112.3 ± 4.7	136.3 ±11.2	116 ± 4.4	125.6 ±13.3	62.4 ± 28.9	(0.000)
	R	69.3 ± 6	86 ± 12.5	128.7 ± 8.1	90 ± 8	93.5 ± 24	63.6 ± 29.7	(0.014)
Elbow1	L	80 ± 2	74.3 ± 5.9	66 ± 2	66 ± 1.7	71.6 ± 6.8	102.4 ± 6.3	(0.000)
	R	39 ± 5.6	56.3 ± 1.2	33.7 ± 3.2	54.7 ± 3.8	45.9 ± 10.7	98 ± 6.7	(0.000)

**Table 8:** The minimum values, *θ*_min_, of the shoulder and elbow angles measured for the Venetian style rowing technique, in degrees, ± 1 s.d., for the individual rowers 1–4, their mean, and its equivalent mean value for standard ergometer rowing, with *p*-value for these two means reported in parentheses.

*θ*_min_		Venetian Rowing Technique (VRT)					Control–Erg	
		Rower 1	Rower 2	Rower 3	Rower 4	Mean	Mean	(*p*)
Shoulder1	L	−17.3 ± 4.2	−9.3 ± 2.9	−2 ± 2.6	−29 ± 0	−14.4 ±10.7	−26.7 ± 9	(0.003)
	R	−0.3 ± 1.5	−8 ± 3.5	14 ± 5	−16.3 ± 2.1	−2.7 ± 12	−27.9 ± 9.3	(0.000)
Shoulder2	L	12.3 ± 2.1	6.3 ± 2.1	9.3 ± 2.5	25.3 ± 3.2	13.3 ± 7.9	18.8 ± 7.7	(0.034)
	R	59.3 ± 4.2	27.7 ± 4.9	44.3 ± 1.2	22 ± 3.6	38.3 ± 15.6	21 ± 5.9	(0.000)
Shoulder3	L	−117.3 ± 4.2	−95.7 ± 5.1	−98.3 ± 8.1	−85.3 ± 7.1	−99.2 ± 13.2	−38.6 ± 34.6	(0.000)
	R	−65 ± 1	−76.7 ±11.6	−83.7 ± 1.5	−64.3 ± 7.5	−72.4 ± 10.4	−42.7 ± 32.6	(0.008)
Elbow1	L	24.3 ± 1.2	24.3 ± 1.5	25.3 ± 0.6	34.7 ± 1.2	27.2 ± 4.6	28 ± 3.6	(0.166)
	R	45.7 ± 2.1	37 ± 3.6	27.3 ± 1.2	42.7 ± 2.1	38.2 ± 7.6	30.5 ± 4.3	(0.008)

**Table 9:** The maximum values, *θ*_max_, of the shoulder and elbow angles measured for the Venetian style rowing technique, in degrees, ± 1 s.d., for the individual rowers 1–4, their mean, and its equivalent mean value for standard ergometer rowing, with *p*-value for these two means reported in parentheses.

*θ*_max_		Venetian Rowing Technique (VRT)					Control–Erg	
		Rower 1	Rower 2	Rower 3	Rower 4	Mean	Mean	(*p*)
Shoulder1	L	46.7 ± 2.1	70.3 ± 1.2	79 ± 4	55 ± 3.6	62.8 ± 13.5	75 ± 3.4	(0.007)
	R	22.3 ± 1.5	46.7 ± 1.2	49 ± 3.6	69.3 ± 1.5	46.8 ± 17.5	72.7 ± 3.8	(0.007)
Shoulder2	L	164.3 ± 3.5	164 ± 2	171.7 ± 2.3	133 ± 2.6	158.3 ±15.7	104.6 ± 28.1	(0.000)
	R	104 ± 3.6	113 ± 5.3	119.7 ± 2.1	104.7 ± 9.3	110.3 ± 8.3	101.2 ± 20.5	(0.000)
Shoulder3	L	20.3 ± 1.5	16.7 ± 0.6	38 ± 3.6	31 ± 3.6	26.5 ± 9.1	24 ± 9.3	(0.568)
	R	5 ± 6.6	9.3 ± 4	45 ± 7.2	25.3 ± 0.6	21.2 ± 17	21 ± 11.2	(0.568)
Elbow1	L	104.3 ± 1.2	98.7 ± 7.6	91.3 ± 2.5	100.7 ± 1.5	98.8 ± 6.1	130.3 ± 7.2	(0.000)
	R	84.7 ± 7.4	93.3 ± 4.7	60.7 ± 4	97 ± 3	83.9 ± 15.4	128.5 ± 6.7	(0.000)

## Data Availability

Data are contained within the article.

## Supplementary Materials

The following supporting information can be downloaded at: https://www.mdpi.com/article/10.3390/bioengineering10030310/s1, Figure S1: Angle-Angle graphs, plotted against Thorax1; Figure S2: Angle-Angle graphs, plotted against LKnee1; Figure S3: Angle-Angle graphs, plotted against LElbow1. Table S1: Detailed statistical information related to angular measurements (in degrees).
