# Supplementary material for: On the Kinematics of the Forward-Facing Venetian-Style Rowing Technique
Source: Bioengineering (Basel). 2023 Feb 28;10(3):310. doi: 10.3390/bioengineering10030310 (PMC10045323; doi:10.3390/bioengineering10030310)
Supplement: Supplementary file 1 [file bioengineering-10-00310-s001.zip › bioengineering-2114540-supplementary.pdf]

On the Kinematics of the Forward-Facing Venetian-style Rowing Technique

by Joseph N. Grima *et al.*

Email: joseph.grima@um.edu.mt; tonio.p.agius@um.edu.mt

Table S1: Detailed statistical information related to angular measurements (in degrees)

Figure S1: Angle-Angle graphs, plotted against Thorax1: (a) Pelvis1 & Hip1 vs. Thorax1; (b) L/RHip1, L/RKnee1 & L/RAnkle1 vs. Thorax1; (c) L/RShoulder1 & L/RElbow1 vs. Thorax1.

Figure S2: Angle-Angle graphs, plotted against LKnee1 : (a) Thorax1, Pelvis1 & Hip1 vs. LKnee1; (b) L/RHip1, RKnee1 & L/RAnkle1 vs. LKnee1; (c) L/RShoulder1 & L/RElbow1 vs. LKnee1.

Figure S3: Angle-Angle graphs, plotted against LElbow1: (a) Thorax1, Pelvis1 & Hip1 vs. LElbow1; (b) L/RHip1, L/RKnee1 & L/RAnkle1 vs. LElbow1; (c) L/RShoulder1 & RElbow1 vs. LElbow1.

Table S1 (Part 1)

Angle*:	Thorax1	Thorax2	Thorax3	Pelvis1	Pelvis2	Pelvis3	Spine1	Spine2	Spine3
**Range of motion, θ_{ROM}**									
Mean	32.3	14.9	14.5	30.4	13.3	14.8	7.0	6.1	5.5
95% CI for Mean (lower)	24.4	11.7	11.6	24.0	9.9	11.8	5.5	5.3	4.5
95% CI for Mean (upper)	40.3	18.2	17.4	36.8	16.7	17.7	8.5	6.9	6.5
Median	31.0	14.5	12.0	30.5	11.5	13.5	7.5	6.0	5.5
St. Dev.	12.5	5.1	4.6	10.0	5.3	4.7	2.4	1.3	1.6
Minimum	15.0	6.0	9.0	12.0	8.0	8.0	3.0	4.0	4.0
Maximum	49.0	24.0	25.0	46.0	23.0	25.0	11.0	8.0	9.0
IQR	24.5	7.8	5.8	15.8	9.3	6.8	4.0	1.8	2.8
**Minimum measured, θ_{min}**									
Mean	39.5	-2.8	-13.4	19.4	-1.0	-10.4	17.6	-2.9	-2.4
95% CI for Mean (lower)	36.3	-6.1	-15.2	15.1	-4.3	-13.4	13.8	-6.4	-5.1
95% CI for Mean (upper)	42.7	0.4	-11.7	23.8	2.3	-7.4	21.3	0.6	0.3
Median	38.5	-3.0	-13.0	20.0	-3.0	-9.0	16.5	-1.5	-3.5
St. Dev.	5.1	5.1	2.7	6.9	5.2	4.7	5.9	5.5	4.3
Minimum	34.0	-10.0	-20.0	8.0	-6.0	-21.0	9.0	-13.0	-9.0
Maximum	49.0	5.0	-10.0	32.0	8.0	-6.0	25.0	3.0	5.0
IQR	7.3	8.8	4.0	10.8	9.0	5.5	11.3	7.5	6.8
**Maximum measured, θ_{max}**									
Mean	71.7	12.1	1.3	49.8	12.4	4.4	24.5	2.9	3.0
95% CI for Mean (lower)	65.3	8.1	-0.2	45.9	8.0	2.2	19.4	-0.7	0.5
95% CI for Mean (upper)	78.0	16.1	2.7	53.6	16.8	6.6	29.6	6.6	5.5
Median	73.0	14.5	0.5	48.5	11.5	4.0	25.0	5.0	1.0
St. Dev.	10.0	6.3	2.3	6.1	6.9	3.4	8.0	5.8	3.9
Minimum	55.0	0.0	-1.0	43.0	3.0	-1.0	12.0	-7.0	-1.0
Maximum	84.0	18.0	5.0	59.0	22.0	12.0	36.0	8.0	10.0
IQR	17.0	10.5	4.8	11.8	12.5	3.5	13.8	10.3	6.8

*Note: *All angles reported in degrees.*

Table S1 (Part 2)

Angle*:	Hip1 (L)	Hip2 (L)	Hip3 (L)	Hip1 (R)	Hip2 (R)	Hip3 (R)	Knee1 (L)	Knee1 (R)	Ankle1 (L)	Ankle1 (R)
**Range of motion, θ_{ROM}**										
Mean	41.0	6.1	7.6	13.7	15.4	11.8	52.4	22.5	34.3	22.9
95% CI for Mean (lower)	33.7	4.6	5.9	9.2	11.1	5.6	43.9	14.6	29.0	17.2
95% CI for Mean (upper)	48.3	7.6	9.3	18.1	19.7	17.9	60.9	30.4	39.5	28.6
Median	45.0	5.5	8.0	12.5	13.5	8.5	54.0	22.0	34.0	20.5
St. Dev.	11.4	2.4	2.7	7.0	6.8	9.7	13.4	12.4	8.2	9.0
Minimum	19.0	3.0	3.0	4.0	7.0	4.0	34.0	7.0	23.0	11.0
Maximum	53.0	9.0	11.0	25.0	28.0	35.0	67.0	41.0	46.0	38.0
IQR	19.0	4.8	3.8	11.5	12.3	10.8	25.8	21.0	16.0	14.0
**Minimum measured, θ_{min}**										
Mean	53.7	-0.3	-6.3	-2.3	-15.3	4.4	6.4	6.7	-38.3	-4.8
95% CI for Mean (lower)	48.8	-3.4	-12.2	-6.0	-21.2	-3.0	-0.3	1.3	-42.6	-13.4
95% CI for Mean (upper)	58.6	2.9	-0.5	1.5	-9.3	11.9	13.1	12.0	-33.9	3.7
Median	56.5	-1.5	-5.5	0.0	-16.5	8.0	3.5	8.0	-41.0	-4.5
St. Dev.	7.7	5.0	9.2	5.9	9.3	11.7	10.5	8.4	6.8	13.5
Minimum	40.0	-6.0	-20.0	-13.0	-26.0	-15.0	-5.0	-13.0	-45.0	-24.0
Maximum	64.0	9.0	5.0	3.0	-3.0	17.0	25.0	15.0	-26.0	10.0
IQR	12.3	7.5	17.8	10.5	19.0	22.5	19.3	10.5	12.3	24.8
**Maximum measured, θ_{max}**										
Mean	94.7	5.8	1.1	11.4	0.3	16.2	58.8	28.9	-3.9	18.4
95% CI for Mean (lower)	86.4	1.5	-5.7	5.9	-4.2	4.5	45.3	18.4	-11.2	12.9
95% CI for Mean (upper)	103.0	10.0	7.9	16.9	4.8	27.8	72.2	39.4	3.4	24.0
Median	100.5	3.5	4.0	7.5	1.5	17.0	54.5	29.5	-4.0	20.5
St. Dev.	13.1	6.6	10.7	8.6	7.1	18.3	21.2	16.6	11.4	8.7
Minimum	75.0	-1.0	-16.0	4.0	-10.0	-9.0	33.0	1.0	-18.0	1.0
Maximum	107.0	18.0	13.0	27.0	11.0	49.0	92.0	54.0	11.0	28.0
IQR	27.0	11.0	20.8	14.8	12.0	32.5	41.3	24.5	21.8	16.5

Note: All angles reported in degrees.

Table S1 (Part 3)

Angle*:	Shoulder1 (L)	Shoulder2 (L)	Shoulder3 (L)	Shoulder1 (R)	Shoulder2 (R)	Shoulder3 (R)	Elbow1 (L)	Elbow1 (R)
**Range of motion, θ_{ROM}**								
Mean	77.0	145.0	125.6	49.4	72.3	93.5	71.6	45.9
95% CI for Mean (lower)	71.5	130.4	117.1	33.5	60.5	78.3	67.2	39.1
95% CI for Mean (upper)	82.5	159.6	134.0	65.3	84.0	108.7	75.9	52.7
Median	79.5	154.5	122.5	47.5	75.0	86.0	69.0	48.5
St. Dev.	8.6	22.9	13.3	25.0	18.5	24.0	6.8	10.7
Minimum	60.0	102.0	107.0	20.0	36.0	63.0	64.0	30.0
Maximum	88.0	166.0	146.0	88.0	94.0	138.0	82.0	59.0
IQR	10.0	38.5	24.3	48.8	32.3	42.0	14.3	21.3
**Minimum measured, θ_{min}**								
Mean	-14.4	13.3	-99.2	-2.7	38.3	-72.4	27.2	38.2
95% CI for Mean (lower)	-21.2	8.3	-107.6	-10.3	28.4	-79.0	24.2	33.4
95% CI for Mean (upper)	-7.6	18.3	-90.8	5.0	48.3	-65.8	30.1	43.0
Median	-12.5	11.0	-98.5	-4.0	37.0	-70.0	25.0	40.5
St. Dev.	10.7	7.9	13.2	12.0	15.6	10.4	4.6	7.6
Minimum	-29.0	4.0	-122.0	-18.0	19.0	-90.0	23.0	26.0
Maximum	1.0	29.0	-79.0	19.0	64.0	-57.0	36.0	48.0
IQR	22.8	13.5	22.3	20.5	30.3	19.3	7.8	15.5
**Maximum measured, θ_{max}**								
Mean	62.8	158.3	26.5	46.8	110.3	21.2	98.8	83.9
95% CI for Mean (lower)	54.2	148.3	20.7	35.7	105.0	10.4	94.9	74.1
95% CI for Mean (upper)	71.3	168.2	32.3	58.0	115.6	32.0	102.6	93.7
Median	64.0	164.0	25.0	47.0	111.0	19.0	100.0	90.5
St. Dev.	13.5	15.7	9.1	17.5	8.3	17.0	6.1	15.4
Minimum	45.0	130.0	16.0	21.0	97.0	-1.0	89.0	56.0
Maximum	83.0	173.0	42.0	71.0	122.0	53.0	107.0	100.0
IQR	24.3	27.3	17.5	34.8	15.0	29.5	12.0	29.5

*Note: *All angles reported in degrees.*

Figure S1(a)

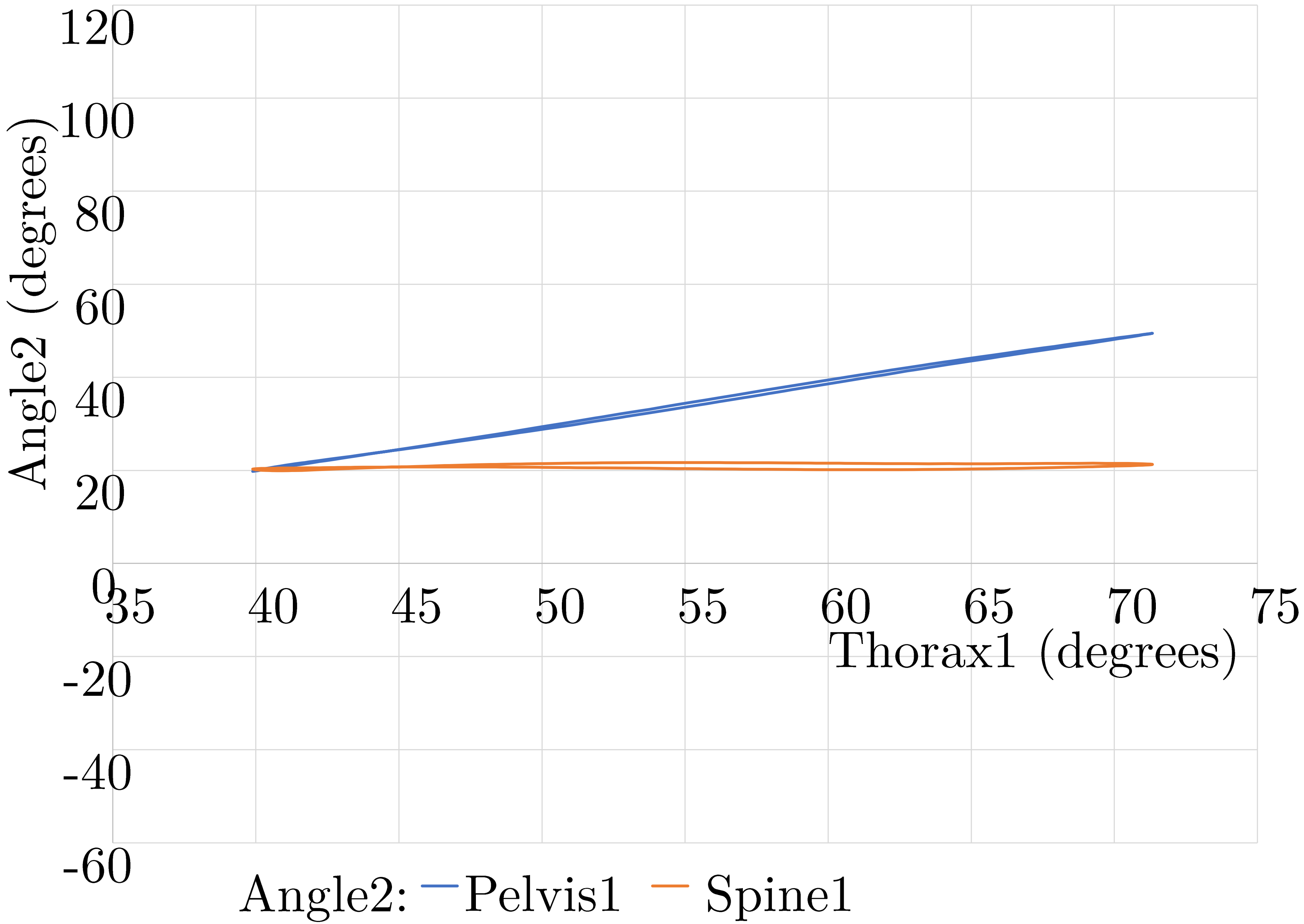

120
100
80
60
40
20
0
-20
-40
-60
Angle2 (degrees)
35
40
45
50
55
60
65
70
75
Thorax1 (degrees)
Angle2: Pelvis1 Spine1

Figure S1(b)

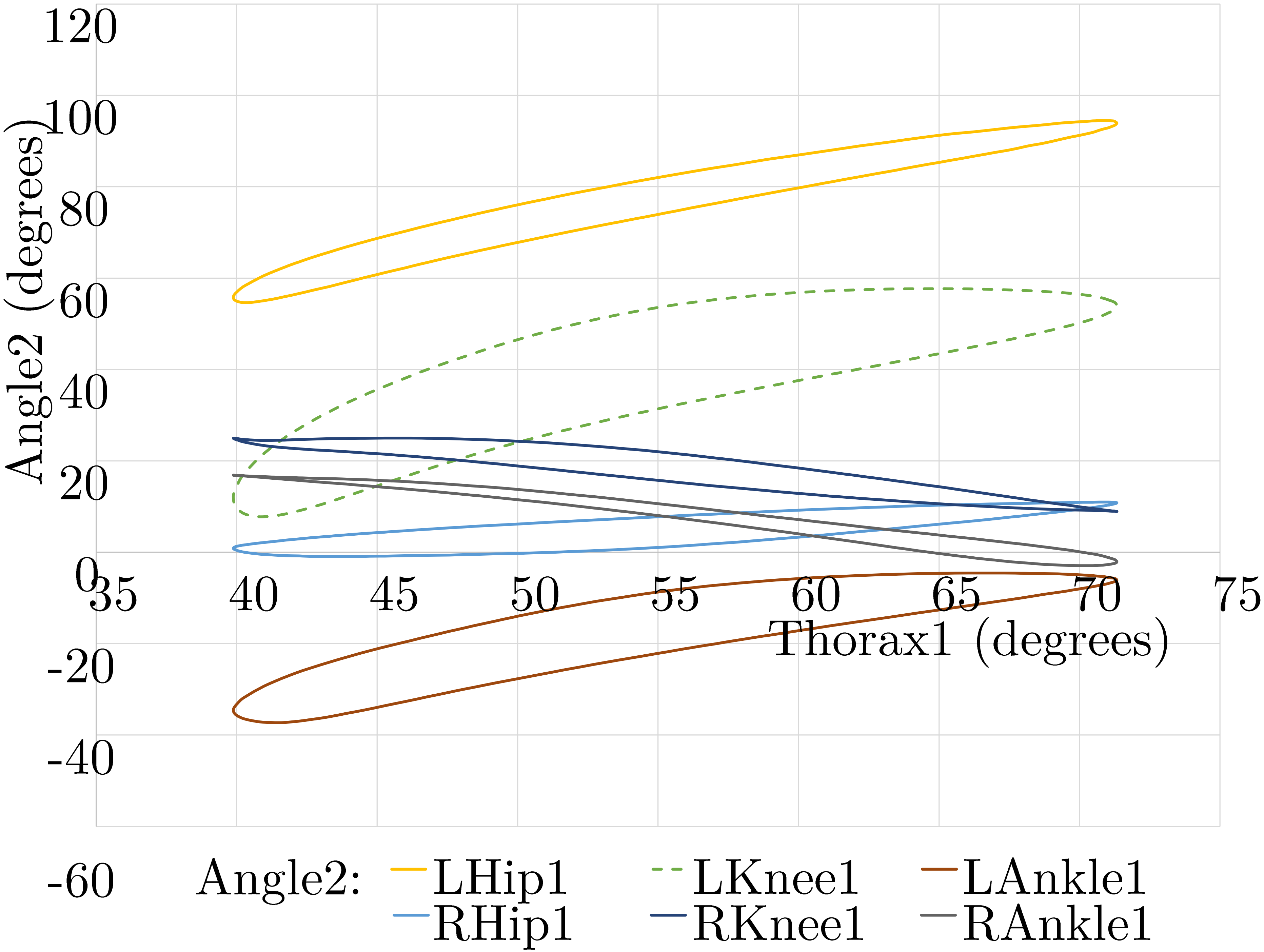

120
100
80
60
40
20
0
-20
-40
-60
Angle2 (degrees)
35
40
45
50
55
60
65
70
75
Thorax1 (degrees)
Angle2: LHip1 LKnee1 LAnkle1
RHip1 RKnee1 RAnkle1

Figure S1(c)

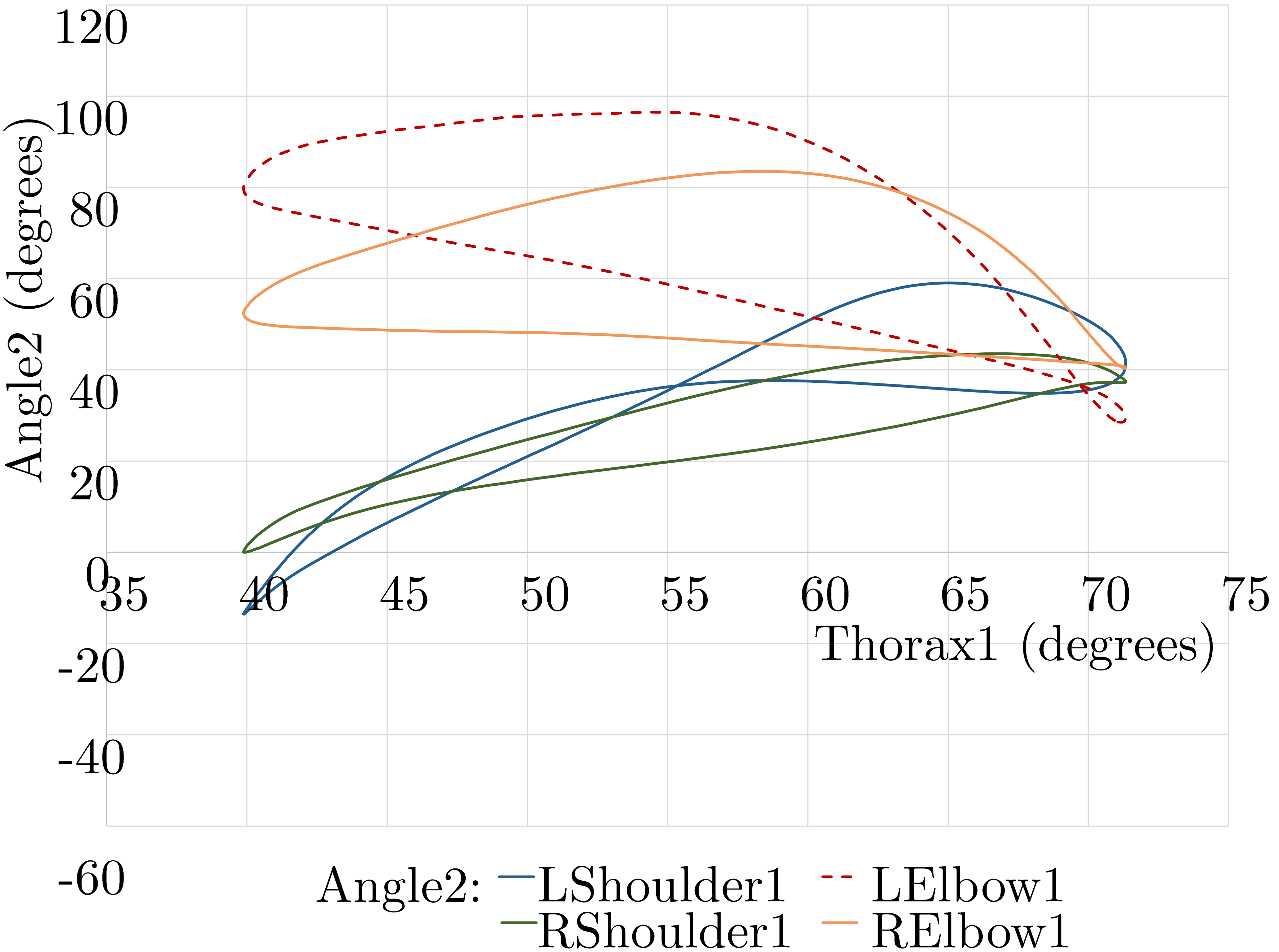
120
100
80
60
40
20
0
-20
-40
-60
35
40
45
50
55
60
65
70
75
Angle2 (degrees)
Thorax1 (degrees)
Angle2: LShoulder1 LElbow1
RShoulder1 RElbow1

Figure S2(a)

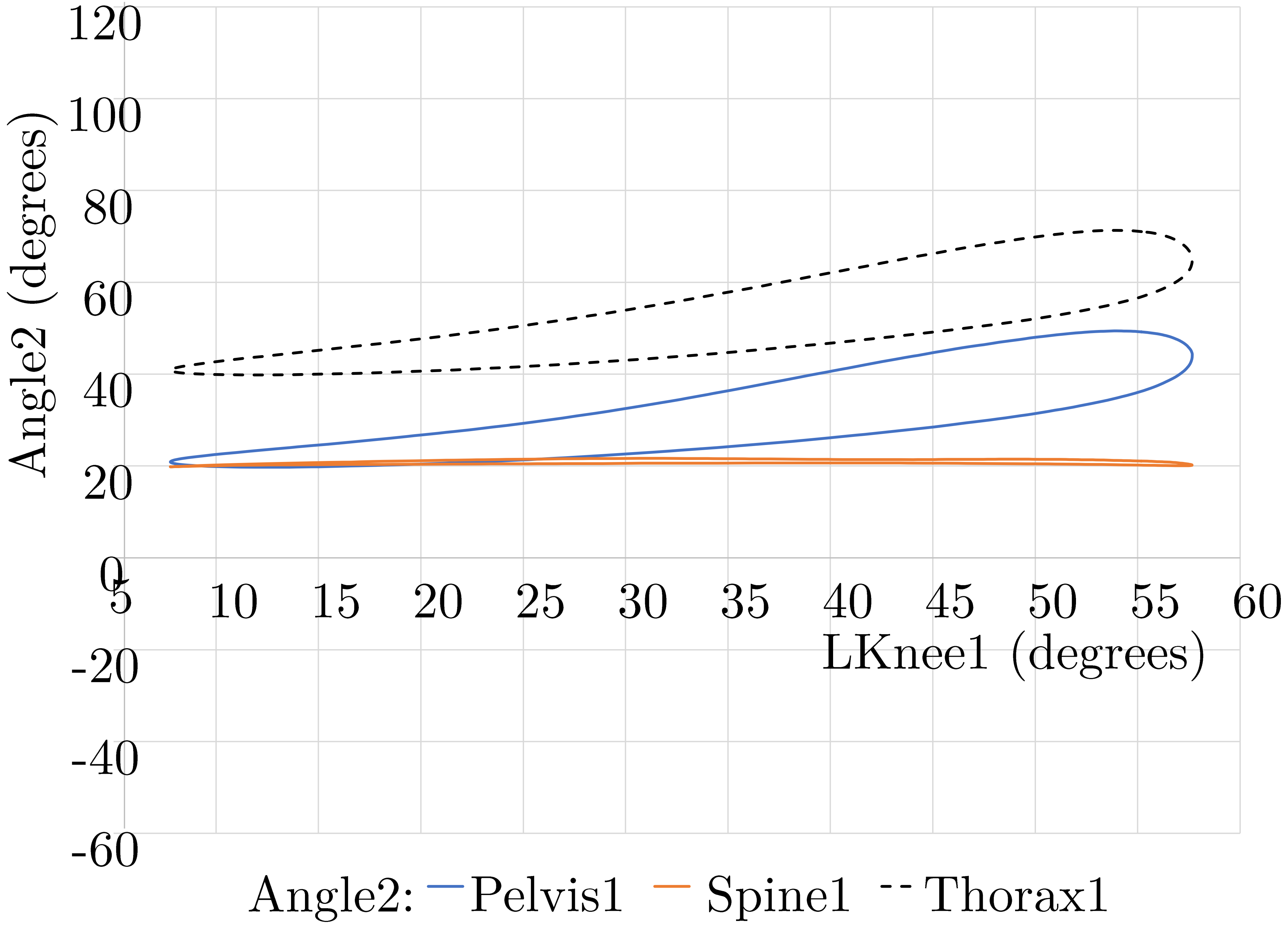

120
100
80
60
40
20
0
-20
-40
-60
Angle2 (degrees)
5
10
15
20
25
30
35
40
45
50
55
60
LKnee1 (degrees)
Angle2: Pelvis1 Spine1 Thorax1

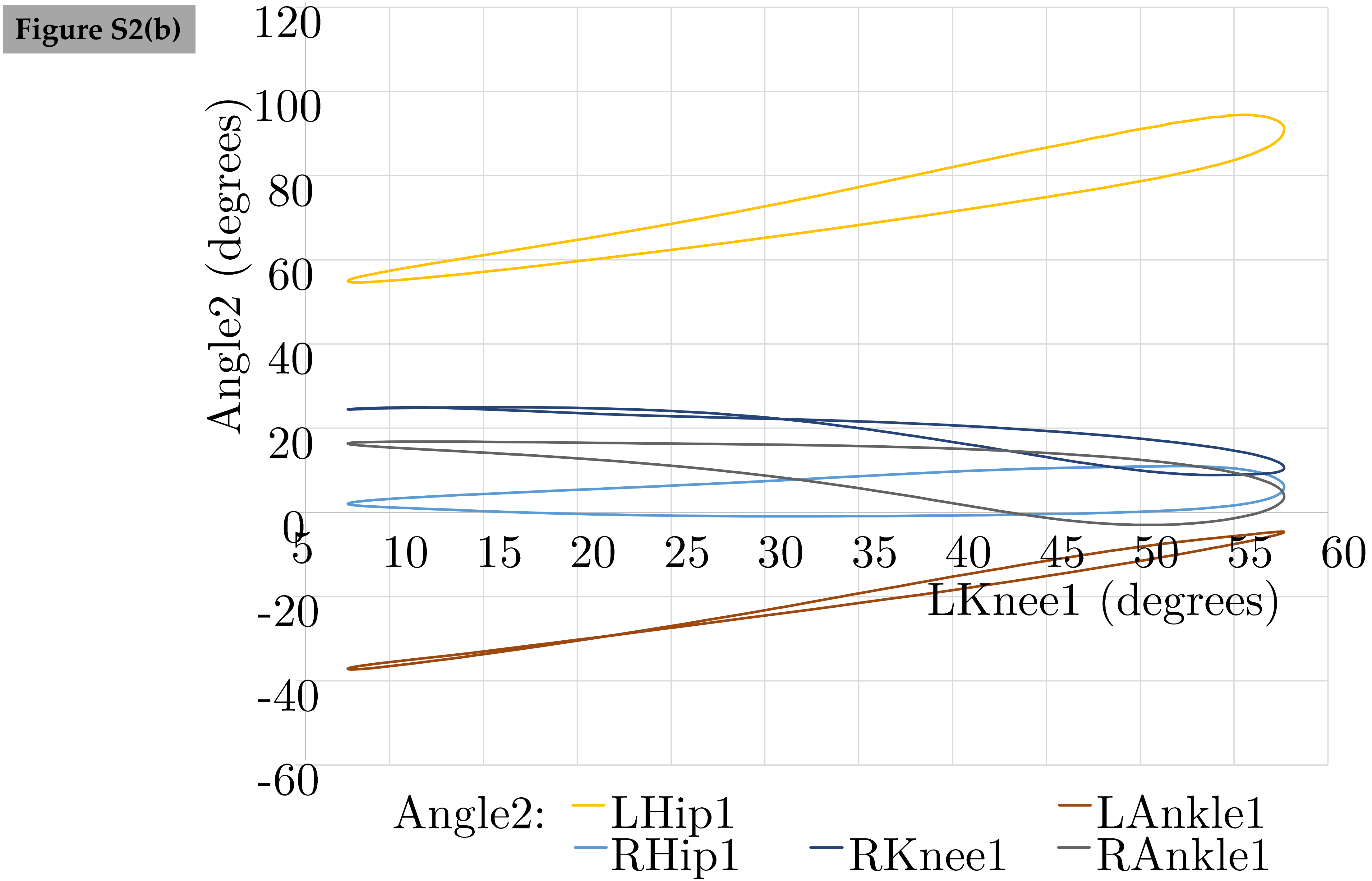
Figure S2(b)
Angle2 (degrees)
120
100
80
60
40
20
0
-20
-40
-60
5
10
15
20
25
30
35
40
45
50
55
60
LKnee1 (degrees)
Angle2:
LHip1
LAnkle1
RHip1
RKnee1
RAnkle1

Figure S2(c)

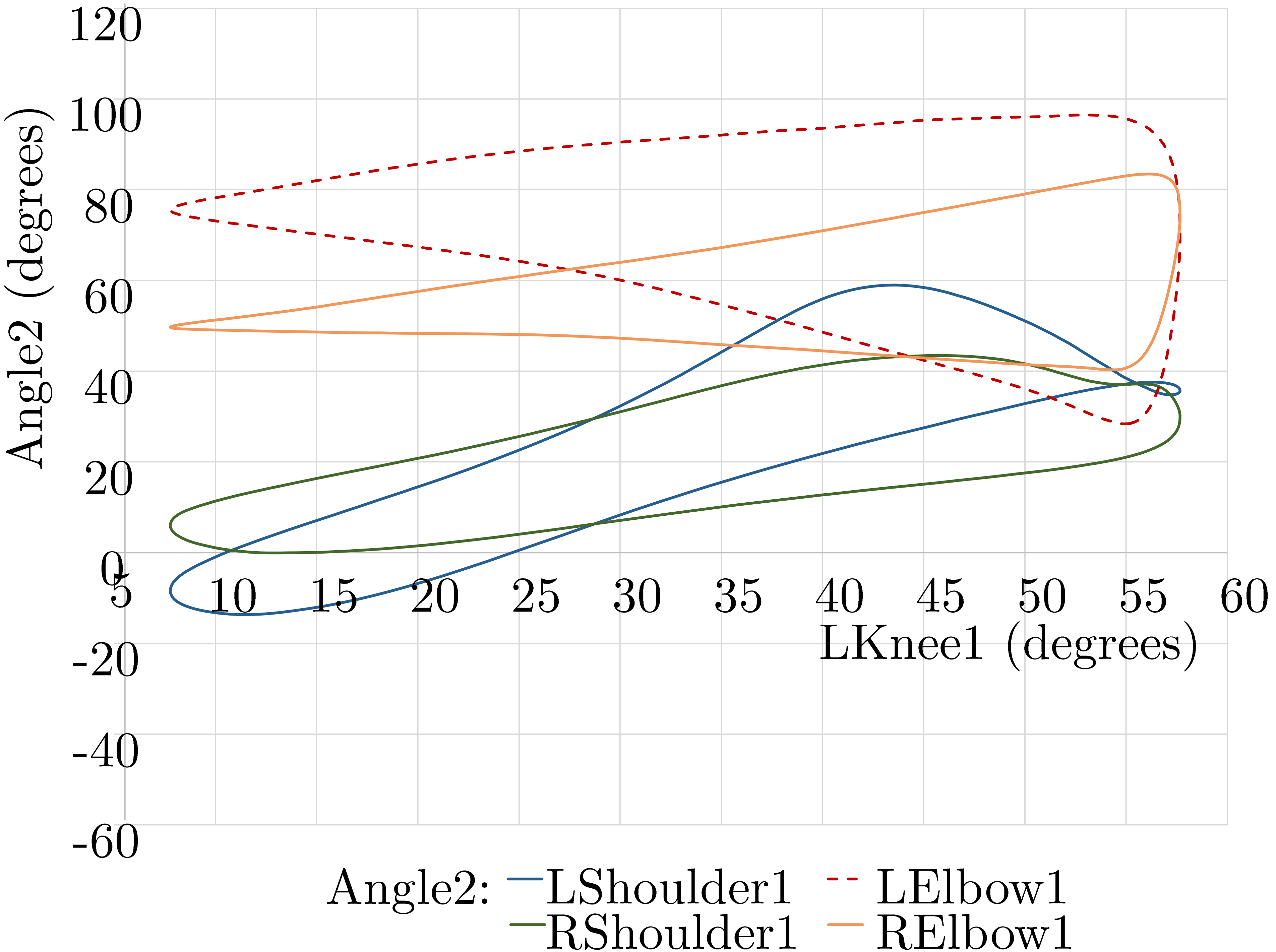

120
100
80
60
40
20
0
-20
-40
-60
Angle2 (degrees)
5
10
15
20
25
30
35
40
45
50
55
60
LKnee1 (degrees)
Angle2:
LShoulder1
LElbow1
RShoulder1
RElbow1

Figure S3(a)

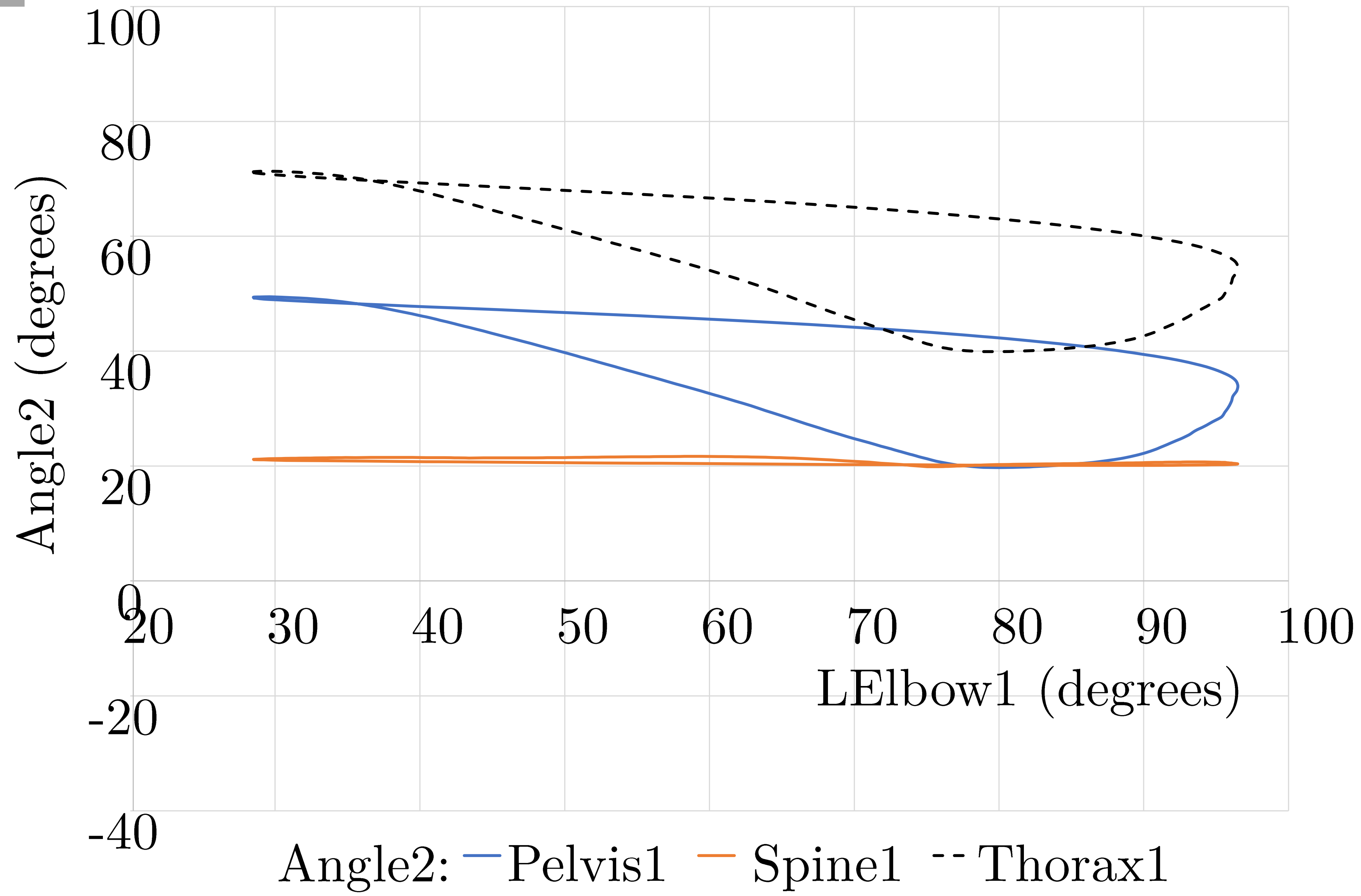
100
80
60
40
20
0
-20
-40
Angle2 (degrees)
20
30
40
50
60
70
80
90
100
LElbow1 (degrees)
Angle2: Pelvis1 Spine1 Thorax1

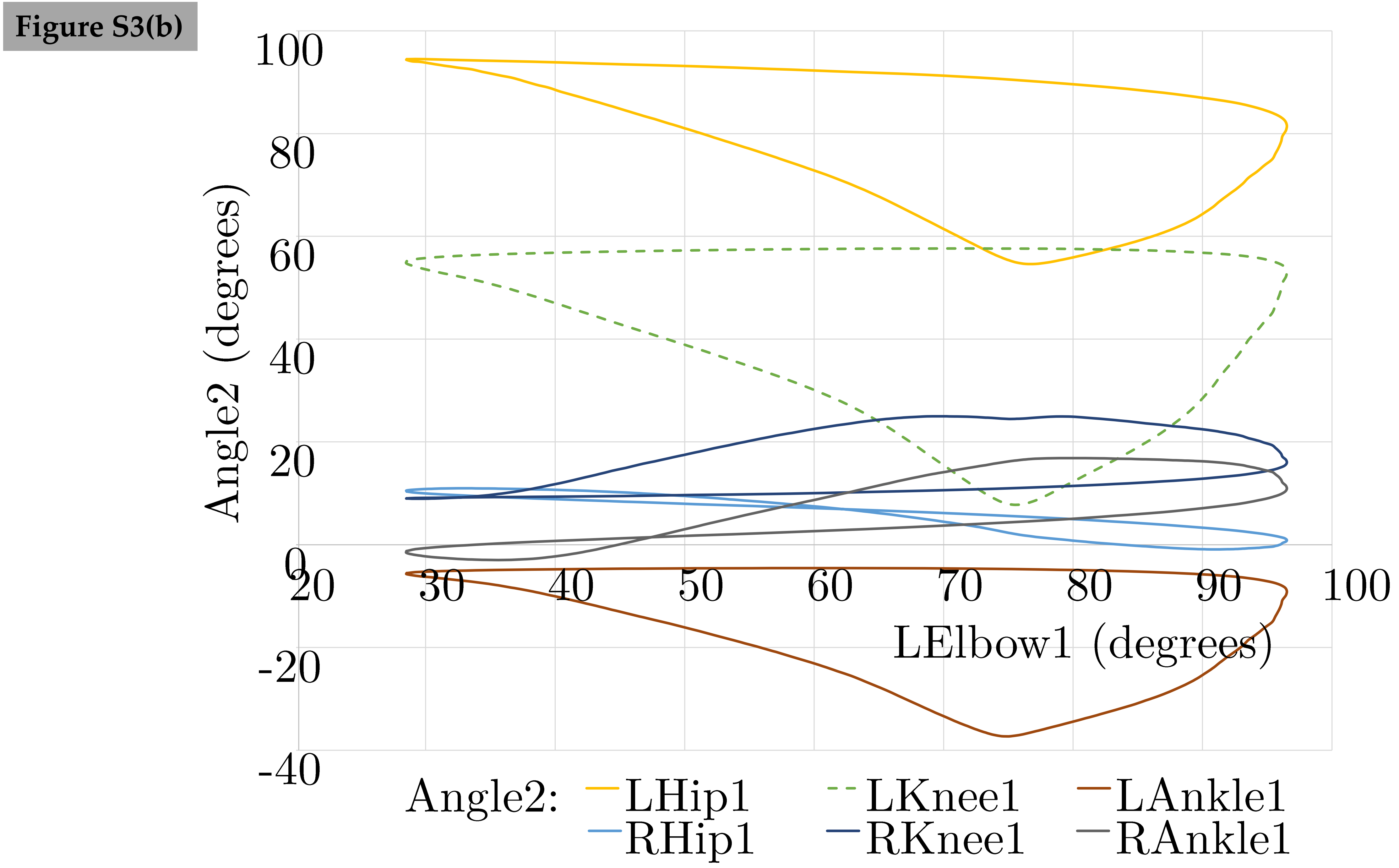
Figure S3(b)
Angle2 (degrees)
100
80
60
40
20
0
-20
-40
20
30
40
50
60
70
80
90
100
LElbow1 (degrees)
Angle2:
LHip1
LKnee1
LAnkle1
RHip1
RKnee1
RAnkle1

Figure S3(c)

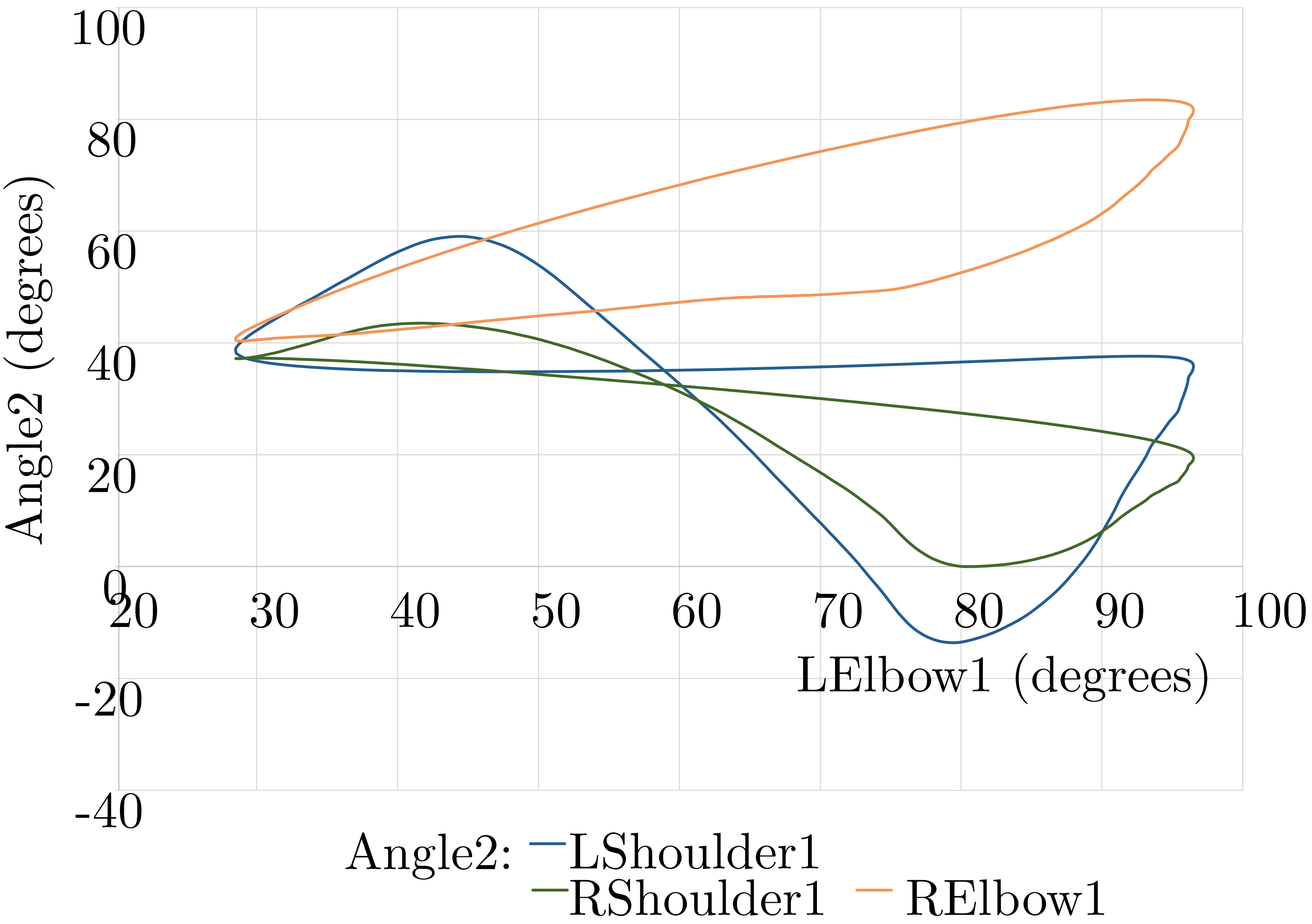

100
80
60
40
20
0
-20
-40
20
30
40
50
60
70
80
90
100
Angle2 (degrees)
LElbow1 (degrees)
Angle2: LShoulder1
RShoulder1
RElbow1
